# Coproducing an Online Platform for People With Long-Term Physical Health Conditions: Development and Usability Study

**DOI:** 10.2196/79666

**Published:** 2026-03-24

**Authors:** Hannah Grace Jones, Grace Lavelle, Elly Aylwin-Foster, Ewan Carr, Alan Simpson, Matthew Hotopf, Vanessa Lawrence

**Affiliations:** 1 Department of Psychological Medicine Institute of Psychiatry, Psychology and Neuroscience King's College London London United Kingdom; 2 Lifelong Health, Persistent Pain Research Group Hopwood Centre for Neurobiology South Australian Health and Medical Research Institute Adelaide, South Australia Australia; 3 IIMPACT (Innovation, Implementation and Clinical Translation) in Health School of Allied Health and Human Performance Adelaide University Adelaide, South Australia Australia; 4 CP-Life Research Centre School of Physiotherapy Royal College of Surgeons in Ireland Dublin, Leinster Ireland; 5 Department of Biostatistics & Health Informatics Institute of Psychiatry, Psychology and Neuroscience King’s College London London United Kingdom; 6 Florence Nightingale Faculty of Nursing, Midwifery & Palliative Care King's College London London United Kingdom; 7 Health Service and Population Research Institute of Psychiatry, Psychology and Neuroscience King’s College London London United Kingdom

**Keywords:** long-term conditions, peer support, coproduction, physical health, mental health, psychosocial support, online peer support, usability testing

## Abstract

**Background:**

There is relatively limited psychological support dedicated to people living with long-term physical health conditions and subthreshold depressive disorder. Online peer support may be an appropriate intervention to help bolster patients’ mental well-being to prevent progression of their symptoms to major depressive disorder. For interventions to be successfully integrated into the self-management routines of people with long-term physical health conditions, they should be co-designed to ensure that they align with the wants and needs of the target audience.

**Objective:**

This study aims to coproduce an online peer support intervention with people with lived experience, software experts, clinicians, and academics through an iterative process of co-design and subsequent co-validation through usability testing.

**Methods:**

We followed a 4-stage coproduction process: co-assess, co-design, co-validate, and co-deliver. Our research advisory group was actively involved in all stages, consisting of 1 coinvestigator and 6 people with lived experience of long-term physical and/or mental health comorbidities. The co-assess and co-design stages involved our participatory design panel, which included 10 members living with various long-term conditions. The participatory design panel participated in online focus groups to assess their unmet psychosocial needs and then co-designed the intervention prototype through online workshops with software developers. The co-validation stage involved an additional group of participants (n=12) with long-term physical health conditions. During co-validation, the prototype underwent usability testing, including think-aloud exercises and semistructured interviews. Content analysis identified the priorities for the iterative development that formed the basis of further research advisory group co-design workshops. The next stage, co-delivery, involved coproducing the protocol of a feasibility and acceptability randomized controlled trial.

**Results:**

Participants highlighted that a platform must feel safe and trustworthy for the space to support the mental well-being of those living with long-term health conditions. The participatory design panel co-designed a platform prototype to meet this need. During the co-validation stage, the think-aloud exercises identified common issues related to navigation challenges and feature glitches. Content analysis of the semistructured interviews confirmed that the community forum, resources, and other platform pages were appropriate and acceptable, but revealed usability concerns. Participants stressed the need for intuitive navigation and suggested new features that would enhance user experience. Facilitators and barriers to engagement were also noted, including the importance of fostering trust in the platform’s ethos and branding to create a safe space. Through iterative development and subsequent usability testing, the final prototype was approved.

**Conclusions:**

We have provided a worked example of a comprehensive, coproduction process where we worked alongside people with lived experience to successfully design an online peer support platform with embedded psychoeducation. The platform, called CommonGround, is ready to be evaluated in a feasibility randomized controlled trial.

## Introduction

An estimated 15 million people are living with a long-term physical health condition in the United Kingdom [1]. Up to 20% are living with comorbid major depressive disorder [2,3], and a further 20% are experiencing subthreshold depressive disorder [4]. Subthreshold depressive disorder is characterized by experiencing between 2 and 5 depressive symptoms for at least 2 weeks, one of which must be either low mood or loss of interest/pleasure [5,6]. Although living with subthreshold depressive disorder is a key risk factor for developing major depressive disorder [7,8], the provision of psychological support to help prevent the worsening of these existing depressive symptoms among people with long-term physical health conditions is relatively limited. Targeted approaches to support the mental well-being of this at-risk population are therefore urgently needed.

A critical component in the effective management of long-term physical health conditions is self-management, whereby patients take an active role in the day-to-day management of their symptoms, treatments, and necessary lifestyle modifications [9,10]. As such, in the development of any tool to support the well-being of people with long-term physical health conditions, it is critical that intervention design and implementation recognize this need to self-manage effectively and should not add undue burden [11,12]. Indeed, empowering patients to address their own needs by providing tools that support their self-management has been associated with improved health outcomes [13,14] and health behaviors [15].

One potential solution to support patients’ self-management of their psychosocial well-being could be peer support, where people with similar life experiences or characteristics (ie, living with a long-term physical health condition) are able to share emotional, informational, and appraisal support with one another to improve their physical and psychosocial health [16-18]. Peer support can be delivered in a variety of formats, from one-to-one to group-based support, and in-person or online. Recently, the provision of online peer support has increased, as it is typically low-cost, easily scalable, and offers a flexible, convenient solution that overcomes the physical barriers people may face in accessing in-person groups [19]. The positive effects of engaging with online peer support resemble those of offline peer support [20], including reciprocal emotional and social support, patient empowerment, improved quality of life, and mental well-being [17,21-24].

Online peer support interventions therefore have the potential to reduce the burden of long-term conditions and the associated psychological distress [16] but also reduce the burden of self-management by improving the tools one has available to self-manage [25]. However, to date, the development of online peer support interventions has largely been driven by academic researchers and industry with limited input from the intended end user [26]. As a result, interventions may not meet end users’ needs and perspectives, and be inappropriately designed, with missing features or poor interfaces [27]. Such poor design can ultimately limit users’ long-term motivation to engage with an intervention, for instance, if they encounter difficulties in trying to log-in [27]. The lack of involvement of end users in design is particularly problematic when the end users are people living with long-term physical health conditions, as this population has complex needs and requires interventions that can be easily incorporated into their existing self-management routines [28]. Indeed, in our prior work, people living with a variety of long-term physical health conditions emphasized that this would be essential to ensure that the intervention feels safe, credible, and appropriate for their needs [29]. Coproduction is therefore vital, whereby an online peer support platform is created through an equal partnership between lived experience experts, researchers, and other partners with the underlying ethos of developing *with* and not *for* the users [30].

Coproduction can enhance the relevance, acceptability, and usability of interventions, thereby facilitating patients’ long-term engagement. The diversity of perspectives fosters innovative and creative interventions, ensuring that features and functions are uniquely designed and rooted in lived experience [27,31]. A critical aspect of coproduction is ensuring the perspectives of lived experience experts have been accurately translated into components of the intervention [27]. One means of ensuring this is by evaluating the usability and acceptability of the co-designed prototype and allowing time for further iterative development as required [32]. Think-aloud exercises are valuable in usability testing for identifying specific usability issues in common user tasks, for instance, glitches encountered when creating an account or creating a forum post [33]. The analysis of think-aloud exercises can also reveal unmet user needs and highlight areas for improvement, guiding future prototype modification [34,35]. To assess acceptability, qualitative interviews are useful as users’ perceptions of acceptability are deeply contextualized (eg, in relation to their long-term physical health condition) and shaped by social and cultural factors [36]. Together, the findings from usability testing can inform the iterative development of the intervention and its ability to satisfy user needs. Ultimately, this process leads to a final approved prototype, which can then undergo real-world evaluation of safety and efficacy in randomized controlled trials.

Although the benefits of coproduction are widely recognized, there is a significant lack of research evidence documenting the coproduction of real-world interventions [37], particularly those dedicated to supporting psychological well-being in the context of physical health [38]. In this paper, we report how we coproduced “CommonGround,” our online peer support and psychoeducation platform dedicated to people living with long-term physical health conditions. This study aimed to coproduce an online peer support platform with embedded psychoeducation for people living with long-term conditions and subthreshold depression to support their psychological well-being. To fulfill this aim, the following objectives were set: (1) to identify the psychosocial needs of people living with long-term physical health conditions, (2) to co-design an online peer support platform prototype that aligns with their wants and needs, and (3) to co-validate the prototype through usability testing and iterative development work to produce a final platform prototype that can be evaluated in a future feasibility randomized controlled trial.

## Methods

### Design

**Figure 1:**
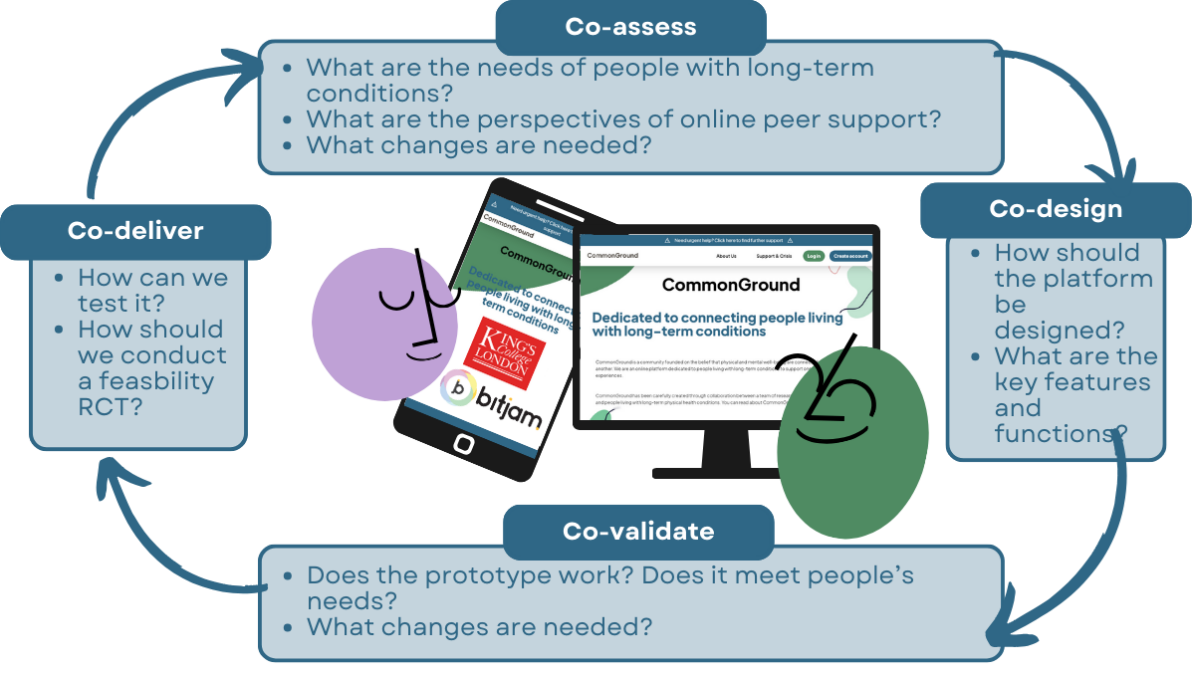
The coproduction cycle of CommonGround, adapted from Elwyn et al [39]. RCT: randomized controlled trial.

The CommonGround project followed a 4-stage coproduction framework adapted from Elwyn and colleagues [39] comprising (1) co-assess, (2) co-design, (3) co-validate, and (4) co-deliver (Figure 1).

### Ethical Considerations

Full ethical approval for the online focus groups and usability testing was sought and granted by King's College London Research Ethics Office, PNM Research Ethics Subcommittee (HR-19/20-14938). Electronic informed consent was obtained before taking part in the relevant research activities (see Multimedia Appendix 1). The procedures to maintain participant confidentiality and anonymity were outlined in the consent forms. All data were pseudoanonymized using unique study IDs, password-protected, and stored on secure servers. Audio-recordings from focus groups and interviews were shared securely with a transcription service, who have signed nondisclosure agreements and deleted audio-recordings immediately following transcription. Participants received a £20 (US $26.9) voucher for participation in the focus groups. The same reimbursement was provided for participation in the usability testing.

### Patient and Public Involvement

**Figure 2:**
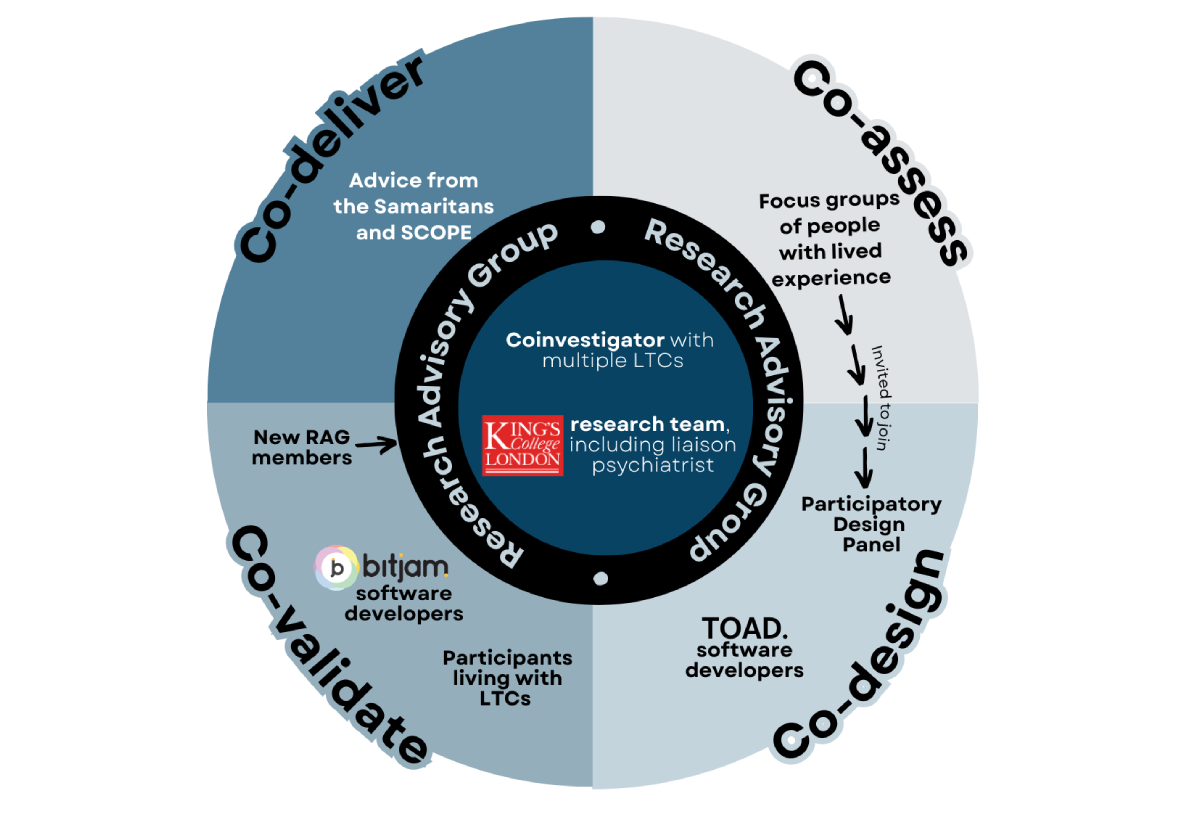
The different stakeholders and groups of people with lived experience involved in the CommonGround coproduction cycle. LTC: long-term condition; RAG: research advisory group.

At each stage of the coproduction cycle, different stakeholders and groups of people with lived experience were involved (Figure 2). One of the authors and a coinvestigator on the project has lived experience of multiple long-term health conditions (cystic fibrosis, type 1 diabetes, and adrenal insufficiency) and, throughout the project, has been a core member of the research team and remunerated for their time. Their role included chairing our independent research advisory group (RAG) of people with lived experience who guided the whole research process, including the coproduction of study documents and key decision-making processes. Our RAG involved 6 members (gender identity: 4 women and 2 men; age range: 31-71 years; ethnicities: Black British, Caribbean, White British, and White European) living with various long-term physical health conditions. Three members were living with multiple long-term conditions, and 4 had comorbid mental health diagnoses. RAG members were identified and invited to join through our coinvestigator with lived experience and through patient representative groups based within Guy's and St Thomas Hospital and King’s College Hospital, and King's College London's Integrated Care Consultation Partners Group, and via snowballing through recruited members. All recruitment materials emphasized the focus on preventing depression in the context of chronic physical illness, and any individual that felt that they had relevant lived experience regarding psychological distress and well-being in the context of their physical health was able to take part (ie, with or without a current or past clinical diagnosis of depression; no formal assessment of depressive symptoms was administered). This inclusive approach not only sought to be representative of a range of lived experiences but was also sensitive to the fact that many people may not recognize that they are experiencing subthreshold depressive symptoms and that these symptoms can also fluctuate over time. Terms of reference were agreed in accordance with UK standards for public involvement [40]. Our RAG members were reimbursed at an hourly rate based on patient and public involvement (PPI) reimbursement recommendations for their time preparing for meetings, attending meetings, and reviewing study documents [41,42]. Members were invited to share feedback outside of any meetings according to their personal preference (eg, via email).

### The Coproduction Cycle

The coproduction cycle had 4 stages: co-assess, co-design, co-validate, and co-deliver (Figure 1) [39], which are outlined below.

#### Co-Assess

The co-assess stage addressed the question: “What are the unmet psychosocial needs of people living with long-term physical health conditions, and how might an online peer support platform address these needs?”

Three online focus groups were conducted, following a topic guide codeveloped with our RAG. Reflexive thematic analysis was conducted using a critical-realist approach [43,44]. The full methodology and findings for this phase have been published elsewhere [29] and laid the foundations for co-designing the online peer support platform. In brief, online focus groups followed a semistructured interview schedule and were transcribed verbatim for analysis. Early stages of the focus group explored their psychosocial needs and how these were linked to their psychological well-being before exploring how these identified needs could be addressed. Participants were asked if, why, and how an online community that connects people living with a variety of long-term physical health conditions would be something that they would use to support the unmet psychosocial needs they identified in the earlier stages. Transcripts were analyzed using reflexive thematic analysis, adopting a critical-realist approach. Following the analysis, the 10 participants who took part in these interviews were invited to be lived experience experts as part of the participatory design panel alongside other stakeholders, for the co-design stage (described below).

#### Co-Design

The co-design stage involved the participatory design panel, which was made up of an external software development agency (TOAD), researchers and clinicians from King’s College London (KCL), our coinvestigator with lived experience, and lived experience experts from the focus groups (n=10) in the co-assess stage [29]. The lived experience experts were originally recruited from King's College London's Integrated Care Consultation Partners Group, the Guy's and St Thomas' PPI group, and the King's College Hospital PPI group. Many were living with multiple conditions, and a range of musculoskeletal, neurological, cardiovascular, respiratory, gastrointestinal, gynecological, autoimmune, and pain-related disorders were represented by the lived experience experts of our participatory design panel. The majority (8/10, 80%) of the lived experience experts used technology daily; however, only 30% (3/10) had used internet support groups before. No formal assessment of depressive symptoms was administered to participants, but recruitment materials emphasized the focus on preventing depression in the context of chronic illness and invited people who felt that they had relevant lived experience of low mood and psychological distress (current or past, with or without a formal clinical diagnosis) to participate. This inclusive, open approach was considered critical in this coproduction process, particularly with regard to identifying a breadth of different perspectives and experiences.

Six online workshops were conducted over 6 weeks, each lasting approximately 2 hours. The KCL team and software developers guided the workshops following specific agendas to maximize the discussions' applicability for creating the prototype (see Table 1). The panel was invited to share any additional suggestions after the sessions via email. The software developers incorporated feedback from each workshop to create the online peer support platform prototype.

**Table 1:** Agenda of the 6 development workshops with the participatory design panel to co-design the platform prototype.

Workshop	Agenda
1: How will the platform help people?	This workshop adopted a “users first” approach. This involved exploring questions such as “What relationships in your life are important to you, and how do you navigate them?” and “Have you looked for information on living with a long-term health condition?”
2: What are users’ needs and wants for the platform?	This workshop focused on assessing user needs in a features-driven approach. This included brainstorming the features and functions that users would like the platform to have. The associated features or factors that may enhance the benefit of and/or reduce barriers of using the platform were also explored.
3: Platform features and solutions	Establishing the core features and how these should be prioritized in the development of the prototype. This involved asking people to rank their preference for different types of social interaction on a platform, from posting content anonymously to commenting in response to other content, and whom they prefer to socially interact with (eg, people with other long-term conditions, whoever is online at that time, and people in close geographical proximity).
4: Psychoeducation and moderation	Identify the type of educational content that the platform should host. Also identify key moderation principles and rules for communication on the platform by asking “What is the platform?” versus “What the platform isn’t?”
5: What is the creative concept of the platform?	Three different creative concepts (eg, color, schemes, and layouts) were presented. Aimed to discuss what will work best and why, to allow further development of the platform features and creative brand.
6: Finalizing the creative bread and prototype	Finalizing the creative brand and prototype, including refining the brand position and name, alongside the visual look and feel.

#### Co-Validate

##### Overview of Co-Validation Stage

**Figure 3:**
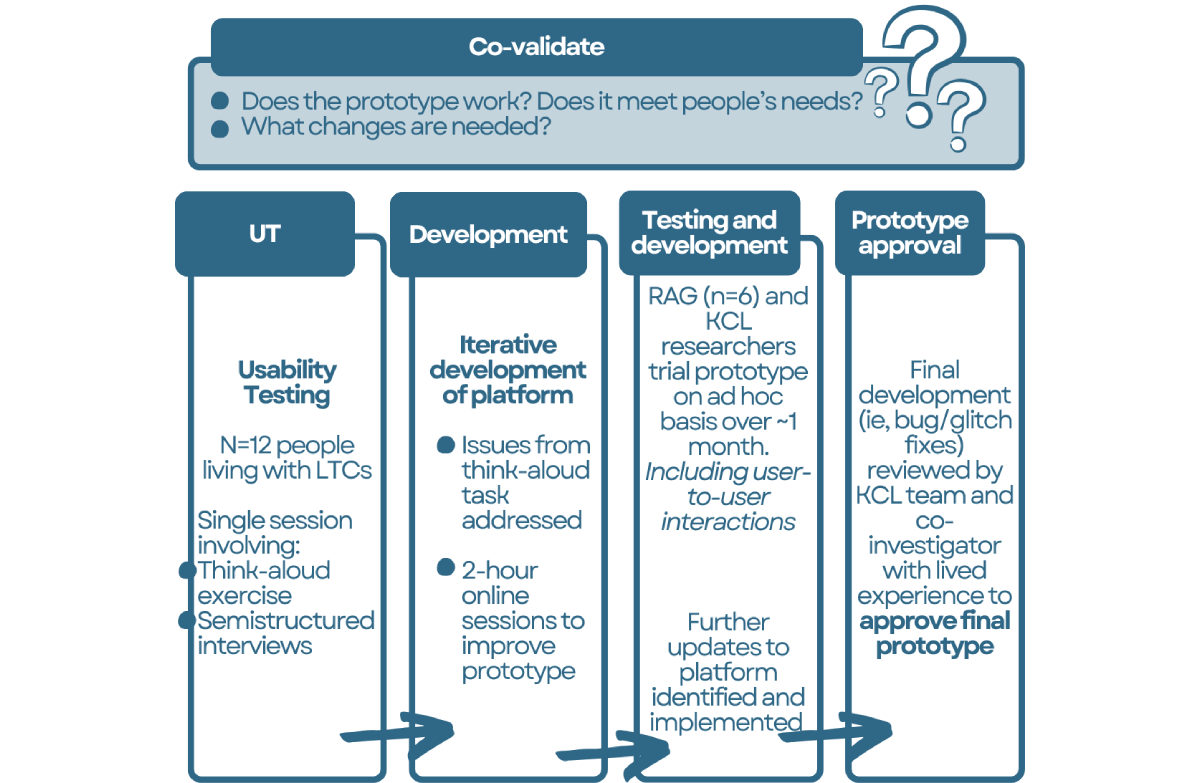
The steps involved in the co-validation stage of the CommonGround project. KCL: King’s College London; LTC: long-term condition; RAG: research advisory group; UT: usability testing.

In the co-validation stage, we sought to review and finalize the platform prototype by answering the following questions: (1) “Have we correctly translated what people wanted into the platform itself?” (2) “Does everything work as we expect it to?” (3) “What, if any, improvements are needed?”. This was an iterative process that involved the following steps (see Figure 3): (1) usability testing: think-aloud task and semistructured interviews, (2) iterative platform development, and (3) further prototype testing, final development work, and final prototype approval.

##### Usability Testing: Think-Aloud Task and Semistructured Interviews

###### Overview

The usability testing involved participants with different long-term health conditions who had no prior involvement in the project. Participants were recruited through national charities and were reimbursed with a £20 (US $26.6) voucher; ethical approval received from King’s College Research Ethics Committee: HR-19/20-14938). The usability testing sessions were conducted via Zoom during the COVID-19 pandemic, as the planned in-person sessions were not possible. Each session included the participant, a research assistant, and a member of the software team. Participants screenshared their view of the platform throughout the session, which involved (1) a think-aloud session (~30 minutes [34]) and (2) a semistructured interview (30-60 minutes).

###### Think-Aloud Session

Participants were asked to verbalize their thoughts as they explored the platform prototype and worked through a series of tasks that a typical user may complete (see Multimedia Appendix 2 for think-aloud activity tasks). For example, they had to create an account, voicing everything that they were thinking as they read through the pages and completed each step. If participants encountered issues in completing a task, the research assistant and software developer encouraged them to troubleshoot themselves, rather than providing explicit guidance on how to overcome the issues.

###### Semistructured Interviews

Participants were then interviewed to explore views on the design and usability of the platform prototype, and potential facilitators and barriers to accessing or using peer support platforms in general. The topic guide (see Multimedia Appendix 3) included questions like “How did you find navigating the platform and finding what you were looking for?” and “Was there anything you looked for on the platform that you could not find?” Interviews were conducted until data saturation was reached: when no new themes or insights were emerging during data collection [45].

All usability testing sessions were audio-recorded and transcribed verbatim. The anonymized transcripts were analyzed in NVivo (version 14, released 2023; Lumivero) using content analysis [46] to organize participants’ views and experiences into 5 predetermined themes and draw realistic conclusions from the data that could be implemented through further co-design workshops to finalize the prototype. Content analysis involved an iterative process of systematically organizing and condensing the data into a coding framework involving the overarching themes of (1) features and functions, (2) usability, (3) branding, (4) barriers and facilitators, and (5) ethos and values. These overarching themes were selected through discussions with our RAG for their ability to organize the data in accordance with the overall objective of coproducing an online peer support platform. Within the themes, codes were developed inductively in response to emerging patterns on a manifest level (ie, taking the meaning of the text at what the text says). Three coders (HGJ, GL, and VL) independently coded 3 transcripts before meeting to ensure consistency in interpretations and make final updates to the coding framework. Once the final coding framework was confirmed, the coders analyzed the remaining data. Bugs and glitches were identified (and coded under a separate code) by extracting statements where participants expressed difficulties or problems when completing each think-aloud task.

##### Iterative Platform Development

First, the software developers from TOAD resolved the glitches and bugs identified in the think-aloud task analysis. The software code was then transferred to the software developers at Bitjam for the remainder of the coproduction. Through a series of 7 two-hour online discussion sessions, the KCL team, our coinvestigator with lived experience, and the RAG agreed on the developments required to improve the existing prototype, and designed the new features and functions that could solve issues or fulfill unmet needs identified in the first stages of usability testing. Based on the RAG discussions, the KCL team prepared user stories and visual mock-ups for all proposed developments to ensure the vision of the RAG was implemented correctly by Bitjam. In these discussions, safety and moderation policies, alongside the related functional features on the platform, were also finalized.

##### Further Prototype Testing, Final Development Work, and Prototype Approval

Following the iterative development of the platform, a review of the refined prototype was completed by the RAG (who had not accessed the “live” platform during development sessions) and KCL researchers (who had no other involvement in this project). This testing occurred over a 1-month period, where testers were encouraged to interact with the prototype as they would with existing social media platforms or forums, logging in on multiple occasions, exploring all the available features, and interacting with other testers. This usability testing aimed to review the new features and functions and identify any bugs or glitches that occur when multiple users are simultaneously accessing the platform and interacting with each other. All testers shared written feedback about bugs and glitches from which the required edits were extracted. The final edits were then implemented by Bitjam. The final prototype then underwent extensive testing by our research assistant and coinvestigator with lived experience before final platform approval was granted by the KCL team.

#### Co-Deliver

Following the successful codevelopment of the peer support platform, the next stages involved coproducing a protocol for a feasibility and acceptability randomized controlled trial. The co-deliver stage involved online group discussions with our RAG and coinvestigator with lived experience to guide study design, the review of study documents, questionnaire testing, and cocreating our adverse events protocol. The Samaritans and SCOPE charity organizations were also consulted with regard to moderation and safeguarding policies, and in the development of moderator training. The protocol of the feasibility and acceptability randomized controlled trial is available elsewhere [47].

## Results

### Co-Assess

The full findings of this work that laid the foundations for co-designing our online platform have been published elsewhere [29]. In brief, findings from 3 online focus groups identified 3 key themes regarding how an online peer support platform could address the unmet psychosocial needs of people living with long-term physical health conditions: (1) relationship between self and the outside world, (2) past experiences of peer support, and (3) philosophy and vision of peer support. Although participants spoke of their mind and body being entwined, they felt this was often neglected in their health care, with their mental and physical health treated as separate entities. The need for their mental health to be supported in the context of their physical health was clear to them. Participants felt that peer support offered both an opportunity to share knowledge and a sense of mutual validation, particularly as people with similar experiences are best placed to understand their circumstances. However, it was clear that online peer support platforms needed to be safe and credible spaces and that to achieve this, platforms must be co-designed with the people who will ultimately use them. Participants also explored the potential benefits and concerns of using online communities, which helped to shape the ethos and philosophy of our platform. Online communities were valued for being easily accessible and easy to participate in; however, it was considered key that a “code of conduct” should be shared and enforced for the space to feel safe and credible. Although many participants were accustomed to the transitions of face-to-face activities to online settings, there were concerns related to how digital interactions may be impaired if equipment or technology does not operate properly. Together, these findings provided the basis for the following co-design stage.

### Co-Design

A prototype of the peer support and psychoeducation platform was successfully developed in the co-design phase with the following features and functions:

- A landing page that introduced the platform
- The community principles that govern the community, presented as “terms of use” that people must agree to before registration
- A registration page, where individuals could create their account and set up their profile
- The community feed which hosted a forum page where users could create posts, add comments, and react to posts
- A resource page where users could view, download, react to, and comment on resources relevant to people living with long-term physical health conditions
- The ability for users to save community feed posts and resources to their private “My Garden” page

The web-based prototype was accessible on desktop and mobile devices. During the co-design phase, the platform name “CommonGround” and the “all diagnoses welcome” ethos were developed. This ethos and related manifesto created by our coinvestigator with lived experience (see Multimedia Appendix 4) reflects the view that people living with any long-term physical health condition have shared lived experiences that they can relate to, from adapting their work or social lives to taking medication and daily self-management. The slogan “share, support, learn, cultivate” was also developed to reflect the main purposes of CommonGround. The term “cultivate” was later replaced with “grow” as our RAG felt this was a more familiar term and better reflected the CommonGround ethos of supporting someone to grow on their self-management journey.

### Co-Validate

#### Usability Testing: Think-Aloud Task and Semistructured Interviews

##### Overview of Findings

The first stage of usability testing included 12 people living with at least one long-term physical health condition (see Table 2) who had not been involved in the co-assess or co-design stages. The range of diagnoses included asthma, chronic kidney disease, diabetes, epilepsy, fibromyalgia, hypertension, multiple sclerosis, myalgic encephalomyelitis, and rheumatoid arthritis. Participants were aged 25-71 years (median 53.5, IQR 29 years), had access to their own internet-connected device, had internet at home, and used the internet daily. Five (42%) participants had used internet support groups before.

**Table 2:** Characteristics of participants involved in usability testing through think-aloud exercise and interviews (N=12).

Characteristics			Values, n (%)
**Gender identity**			
	Woman, including transgender woman	11 (92)	
	Man, including transgender man	1 (8)	
**Number of participants living with multiple long-term conditions**			
	1 long-term condition	5 (42)	
	2 long-term conditions	4 (33)	
	3+ long-term conditions	4 (33)	
Number of participants living with comorbid mental health conditions			3 (25)
**Do you have access to the internet at home?**			
	Yes	12 (100)	
	No	0 (0)	
**How frequently do you use the internet?**			
	Daily	12 (100)	
	Weekly	0 (0)	
	Monthly	0 (0)	
**Devices used to access the internet? (more than one device could be selected)**			
	Laptop	7 (58)	
	Desktop	1 (8)	
	Phone	7 (58)	
	Tablet device	3 (25)	
**Have you used internet support groups before?**			
	Yes	5 (42)	
	No	7 (58)	

From the think-aloud task, explicit verbal indications of bugs and glitches were successfully identified and consolidated (see Table 3 for full results). In total, 9 usability issues were identified. The most common usability issues were related to navigation, layout, understanding instructions, and the multiple steps required to complete a task. The most usability issues were encountered for the “create an account” task. Across ages, gender, and long-term physical health conditions, experiences of issues were similar, with the majority of issues experienced by most participants.

**Table 3:** Think-aloud tasks and usability testing results for each task.

Usability issues		Description of issues	Example Quotes
**Task 1: browse around for 1-2 minutes, providing your feedback [logged out state]**			
	Navigation	Users had difficulty moving around the home page. Some experienced a sticky scroll. The arrow at the bottom of the page that intended to move the user down did not function properly.	“So, basically, it doesn't scroll. It doesn't scroll so easy. I think when, when I put in my cursor on the blank space on the side, then it tends to scroll a bit. So, it's a bit erratic, the way it scrolls.” [Participant 1] “Once you click to go back up it’s, kind of, doing that thing that if you try and go to go down with the arrow it, kind of, keeps doing that jump thing.” [Participant 8]
	Layout	Users could not read all the text as some was obscured or not displayed properly.	“See, I can’t read, I can’t read that because the pages kind of separates, and you’re kind of missing a little bit of something there.” [Participant 1] “The first thing I've noticed, I can't read the top line there.” [Participant 6]
**Task 2: create an account**			
	Navigation	Users had difficulty in identifying the button to create an account, often selecting the wrong button.	“Right, so where, where do I go to join an account, to make an account?” [Participant 2] “Where do I create an account? Or do I have to login? Right. So, do...? Am I using? Do I put me email address in and stuff?” [Participant 3]
	Understanding instructions	Users were confused about what to input for various fields of the registration form and desired further instructions.	“I think what's really interesting there is it wasn't really clear for you where to start. It's the question’s so broad. So, we can look at, you know, maybe having a couple of kind of tips underneath or something.” [Participant 1]
	Glitchy function	Users struggled with the calendar date selector when inputting date of birth.	“That was a bit tricky writing down a date of birth... but I did about...I think it was the third time I typed it in. Like I said, here I think a drop-down menu would be easier.” [Participant 7] “Mm, don’t even know how to put the date in.” [Participant 11]
	Workflow	Users often missed a step in the registration form, having to review closely to resolve the issue and proceed.	“What’s that? I’ve got a hand, so that’s saying that’s a live link, but it’s not allowing me to (click)...Oh, okay. So I’ve gotta put something else then?” [Participant 7]
**Task 3: browse around for 1-2 minutes, providing your feedback [logged in – community feed]**			
	Visuals	Users were unsure what some of the graphics and labels of buttons meant, and it created confusion about the potential functions of different buttons.	“I’m not sure what the Add to Garden means. I might be a bit dumb [laughing] if I don't know, but I’m not sure, um, about that, if I’m honest.” [Participant 12] “The, you know, the things you have, the images that you have above the post a thought, share knowledge, ask a question, I was just wondering what that is, what those images depict.” [Participant 1]
**Task 4: navigating between pages for tasks: (1) find a psychoeducational resource and view it and (2) view posts from other community members**			
	Navigation	Users often did not notice the navigation bar as it did not stand out on the page. This limited their ability to switch between pages, including to find a resource or to go view their “My Garden.”	“Okay. Okay, so it took me a while to see that thing at the top where I’ve got the options for, Community Resources In “My Garden”. Um, I didn’t see that until I’d scrolled all the way down to the bottom.” [Participant 11] “I think it’s because I completely didn’t realise that those were up there. If I maybe did. Maybe if they were bigger. I’m, I’m trying to...I don't know what other things you could do to, um, make it more noticeable.” [Participant 12]
**Task 5: create a post**			
	Workflow	Some users had trouble in completing the different fields for creating a post.	“Mmm. I don’t know. Is there a way to, sorry, I don’t know how to work this. Is there a way to like, type to here, or...” [Participant 4]
**Task 6: view posts from other community members**			
	Navigation	Some users did not know how to comment on a post.	“I’d be looking for a, um, respond button to be able to get back to [username], you know?” [Participant 10]

The content analysis of the usability testing transcripts grouped the data into the 5 themes predetermined from the agendas of the co-design workshops. The first 4 themes—features and functions, usability, branding, and barriers and facilitators—are presented in full in Table S1 in Multimedia Appendix 5. Table S1 in Multimedia Appendix 5 also describes the platform modifications and subsequent development work that were considered and/or implemented to address participants’ feedback. The fifth theme—ethos and values—is depicted in Table S2 in Multimedia Appendix 5. Within each theme, codes and subcodes were also added as appropriate during analysis (see Tables S1 and S2 in Multimedia Appendix 5).

##### Theme 1: Features and Functions

This theme included the codes of additional features, content and materials, community page, “My Garden,” and joining form. In addition to the bugs and glitches participants experienced when creating an account, upon reflection, some expressed apprehension about signing up and agreeing to the community principles (terms of use). For instance,

It's almost like these are all our rules and regulations, and you have to agree to all of these rules and regulations before we will even tell you anything. That was, that put me on my guard, really.

Participant 10

Participants expressed the need to fully understand “what am I agreeing to,” suggesting that background information about CommonGround and who owns it is needed to help people decide whether to join. However, participants shared that the personal information required matched their expectations of what creating an account typically involves. Throughout the feedback, it was clear that making the account creation process as easy and as informative as possible was important for facilitating registration on CommonGround.

To be honest, it’s, it’s really straight forward and it kind of makes it easier because it’s, it goes along with what the majority of the registered, sort of, websites go for.

Participant 8

Participants generally had positive views of the community, resources, and “My Garden” pages. Specifically, people appreciated the unique features of the community forum, stating that the different types of posts you could create were “something I’ve not seen ever before” [Participant 2], and that the “reactions are great... the ‘I hear you’ and this (thinking of you) is helpful, it’s quite nice to see that” [Participant 8]. Some participants were unsure what the “My Garden” page was for, but were keen to use it once they understood that they could save posts of interest to their own private “garden”: “that really appeals to me, because I can, I can, yeah, grow my own library, oh, that’s heaven [laughs]” [Participant 12]. Participants suggested that additional text should be added to the “My Garden” page, and the “save post” button on forum posts should be changed to “add to My Garden,” to clearly demonstrate how the page can be used.

Participants also shared the view that new additional features could improve the platform. For instance, participants expressed a clear desire for a search bar to fulfill their need of easily finding specific content of interest and overcoming issues in navigating the platform: “I would seriously just give me a search bar and let me write in what I want to look for” [Participant 10]. Participants also suggested adding pages that share “about us” information and the full moderation policy.

##### Theme 2: Usability

This theme included the codes of platform navigation, visual design, visual layout, and tone and language. Overall, participants indicated that the platform “is quite user-friendly” [Participant 3], and that they felt comfortable using the platform, even if they did not feel technologically adept themselves:

I wouldn’t say that I’m fantastic in technology. So, it was...It’s, it’s...I think it’s quite technically friendly, and quite easy to access, to be honest.

Participant 9

Participants stressed the importance of the platform being intuitive to navigate with a typical website layout. For instance, participants expected there to be a menu bar as they have “got so used to seeing a menu thing at the top that when I go on to any website, that’s the first thing I look for, is that menu” [Participant 10]. Although the prototype had a menu bar, participants “didn’t initially notice that they (the menu bar and page tabs) were there at all” [Participant 12], until asked to complete a task involving switching to a different page that forced them to search around the page for it.

Participants appreciated the simple, clear, uniform layout of the community feed, with posts that are “a nice size to be able to digest” [Participant 11]. While many participants liked the colors and graphics of the platform, some of the graphics used to indicate the functionality of buttons were not sufficiently clear: “the images that you have above the post a thought, share knowledge, ask a question, I was just wondering what that is, what those images depict” [Participant 1]. Participants also wanted some clarity on the language used within the site copy, particularly for terms relating to peer support and mental health that may be unfamiliar:

Peer to peer...I think at times, it could be slightly wordy...It’s a term that I've come across, yes definitely. I'm not sure it’s term that everybody will know, though.

Participant 1

Participants also shared that extra explanations would be helpful on the resources page, when introducing the resources overall (“Um, you say end, endorsed by experts...But who, who exactly?” [Participant 2]) and on specific topics such as “keeping active” (“I’m a little bit confused on when you say active, is that more like exercising keeping active sort of thing or...?” [Participant 8]).

##### Theme 3: Branding

This theme related to how participants felt an online platform should be presented to the world, including the codes of the platform name, platform branding, and advertising. Participants felt that the name “CommonGround” clearly communicated a safe space for people who have the shared experience of living with long-term health conditions to connect. However, participants were unsure whether the name was able to communicate the fact that the platform has a focus on supporting mental well-being:

I do think that’s a really good name, but, um, I don’t know whether it says enough...It depends how people are introduced to CommonGround...If it’s something you find, you know, if you Google, if you Googled psychological services, and CommonGround came up, then I don’t think it would really matter.

Participant 8

These views were also expressed in relation to the logo:

I know CommonGround is probably the main logo. Um, reading CommonGround for the first time you wouldn’t exactly think it’s, like, health-related or anything.

Participant 6

Having a brand, including the name and logo, that communicates the purpose of CommonGround was suggested to facilitate engagement with the platform.

The importance of being able to trust the online platforms was emphasized. Participants wanted to know who owns the platform and if their motivation is to genuinely improve the well-being of users or to generate profit. In the case of CommonGround, the KCL logo on the landing page helped develop trust in our motivations and governance: “you’re under King’s College so that gives you that credibility anyway” [Participant 11]. Participants suggested that advertising the platform through avenues that people with long-term conditions trust, including their health care teams or charities, would also foster trustworthiness in CommonGround:

I think it carry, it would carry more weight. You know, if a, if a professional has recommended something.

Participant 2

##### Theme 4: Barriers and Facilitators

During their reflections of exploring CommonGround, participants identified some barriers and facilitators that might affect someone’s interest in, and engagement with, a peer support platform. For instance, someone’s desire to use the platform might be limited by their perceptions of peer support, and whether peer support is suitable for them or how to engage with it appropriately: “(being) on this platform practically every day. That’s not healthy” [Participant 7]. For those who were motivated to use CommonGround, participants believed that a user’s technological competency would not be a barrier. Participants who self-identified as “not very tech-savvy” felt “like it’s very open to a lot of people” [Participant 8] and “it’s quite technically friendly, and quite easy to access” [Participant 9]. However, there were concerns related to the safety of the online community, which may be vulnerable to the sharing of misinformation or malicious links, and infiltration by rogue users:

It's just you know how there's like a lot of spam, a lot of scams, especially when it comes to links sort of thing. And you never know what you're clicking on. So, somebody who's got good intention and coming on the website, you don't know if that's going to be everybody's intention.

Participant 8

##### Theme 5: Ethos and Values

The ethos and values theme had codes including platform purpose, the meaning of coproduction, and the power of peer support (see Table S2 in Multimedia Appendix 5). The fact that CommonGround was rooted in coproduction was considered essential, with participants emphasizing the importance of combining lived experience alongside academic experts who bring scientific rigour:

Co-design is essential if you’re doing anything for a, a particular audience. You, you, you must include representatives of that audience if you want to get a, a, a fully rounded, um, end product.

Participant 7

That CommonGround would fulfill a current gap in support for people with long-term conditions was also noted, with participants sharing that CommonGround is “much needed” [Participant 1], particularly for its dual focus on the mind and body: “I mean, as well as your physical health conditions, I think it would help tremendously with my mental health” [Participant 6].The power of peer support was recognized through participants’ previous experiences, where the ability to connect anonymously with people “like me” is powerful, as sometimes there was hesitancy to speak with people in their existing social circles:

A lot of people I, I, I would imagine would, would talk to somebody on the bus or whatever, before they’d talk to family, or friends even...The good thing about that is nobody knows who I am.

Participant 5

Although sharing experiences can help alleviate feelings of “why me,” some concerns were shared of becoming overfocused on the effects of their long-term conditions during these exchanges, when they might rather “just get up and get on with my life” [Participant 7]. Participants recognized that a peer support platform can be beneficial at times, but also something to take some space from at other times dependent on their personal circumstances and well-being needs.

#### Iterative Platform Development

Table S1 in Multimedia Appendix 5 outlines the platform development opportunities identified through content analysis of the think-aloud exercise and interviews that guided the iterative development process. The software developers were successful in completing the development work agreed by the RAG and KCL team. For instance, in response to the need for improved navigation, the RAG designed a search function. This included adding a search button within the navigation menu and allowing users to search by free text or content tags (similar to hashtags), with the option to filter results by recency or relevance.

Throughout the iterative process, the RAG also created additional features to enhance user experience. One key addition was the “How I Am Feeling Now” feature. The RAG recognized the importance of being able to communicate one’s emotional context in forum posts to help their peers understand the type of support they are looking for. The RAG therefore suggested that when users are creating their post, there should be an option to select an emoji that represents their current emotional state or mood which then appears alongside the post’s text to give this emotional context.

#### Further Prototype Testing, Final Development Work, and Final Prototype Approval

The testing by the RAG and KCL volunteers identified minor bugs, slow loading times, and that notifications were not being sent or received properly. Other feedback included suggestions to reorder or reformat text, for instance, by making key information more prominent or highlighted to catch the attention of end users who may skim through the content. The final required edits were successfully implemented, with the final prototype for the feasibility randomized controlled trial completed in May 2024. Screenshots of the final platform prototype are provided in Multimedia Appendix 6.

## Discussion

### Principal Findings

There is a current lack of psychological support to help people with long-term physical health conditions manage their mental well-being that is easily accessible and tailored to their specific psychosocial needs, particularly for those at risk of developing major depression [48,49]. In response to this gap in psychological support, we sought to coproduce an online peer support platform following a 4-stage, iterative coproduction cycle. While coproduction has become the gold standard for intervention development [28], to date, end users have rarely been involved in all aspects of intervention design, development, and evaluation [27]. Our work serves as an exemplar of how to coproduce an online peer support and psychoeducation platform by creating partnerships between people with long-term physical health conditions, academics, clinicians, and software experts. In the co-assess stage, people living with long-term physical health conditions expressed that an online intervention that provides a safe and trustworthy space for connecting with others like me, while acknowledging the mind-body interplay, could support their psychosocial well-being [29]. Following the co-design phase, which resulted in the successful creation of an initial prototype, usability testing during the co-validation stage confirmed that CommonGround included features and functions that were appropriate and acceptable. Critically, areas for improvement were also identified, such as making platform navigation more intuitive, resolving platform glitches, and building new features to enhance user experiences. Ultimately, through the iterative coproduction cycle that consistently places the voices of lived experience experts at the foreground, we created an intervention that has been specifically tailored to our target audiences’ unique characteristics and needs, from the specific features and functions to the “all diagnoses welcome” ethos and platform governance. The CommonGround platform is now viable and ready to be evaluated in a future feasibility randomized controlled trial.

As noted in the development of other mobile health applications (eg, [50]), we found that iterative usability testing was critical for resolving lingering bugs and glitches to minimize the impact of these problems on user engagement [51]. Notably, many of the usability issues our participants experienced are commonly encountered during usability testing, such as navigational barriers and errors in site copy [52]. Our participants’ emphasis on the importance of CommonGround being easy to understand and intuitive to navigate offers further evidence of the critical role that language and usability play in facilitating engagement with peer support platforms [16]. This finding also aligns with the technology acceptance model, which states that whether a user will accept or reject an online platform is partially dependent on how easy it is to use [53]. Together, these insights highlight the importance of incorporating structured, iterative usability testing throughout the coproduction of novel online interventions to ensure that they are technically functional, accessible, and aligned with the anticipated end user’s needs.

An individual’s digital literacy is often cited as a potential barrier to engaging with online interventions [19], yet interestingly, our participants did not identify technological competency as a potential barrier to accessing CommonGround. As participants described the platform as relatively intuitive to navigate, this could suggest that CommonGround is easy to use and does not require a significant level of digital literacy. However, it is important to note that although some participants identified as “not very tech-savvy,” they were all daily internet users and may not represent the views of those with limited internet access or digital experience. This highlights the importance of actively recruiting people who may not typically engage with online interventions to comprehensively explore potential barriers to using online peer support forums [16].

Developing an online intervention, however, goes beyond creating the features and functions that may improve end users’ experiences. For instance, the importance of CommonGround being a safe space, and underpinned by consistent, reputable, and trustworthy branding, was emphasized throughout the co-design and co-validation stages. This echoes prior work highlighting how the inclusion of reputable branding, logos, and declarations of ownership can positively influence the trustworthiness and credibility of web-based health information [53,54]. Another facet of safety that our participants highlighted was technological security. Concerns about security and data protection are commonly cited as barriers to engaging with online interventions [55], with older users, those from high-income backgrounds, and those who had a previous experience of a data breach expressing the most apprehension [56]. This highlights the importance of collaborating with software experts to build a robust, protected online interface with security features to minimize potential threats, assure data confidentiality, and maintain privacy. However, given that concerns about security vary between users, it is pertinent to develop security features alongside lived experience experts to strike a balance between allaying fears (a facilitator to engagement) and avoiding excessive measures (a barrier to engagement) [57,58].

The “power of peer support” emanated as a central theme throughout our coproduction process, reinforcing previous findings among people with long-term conditions that online peer support provides a valued space to strengthen social ties [21] and can improve an individual’s emotional self-management and self-efficacy [16]. Throughout this work, our PPI and research participants echoed these findings, emphasizing the unique value of peers in providing valuable information and support, independent of their existing social ties [16,59,60] and reducing loneliness by providing connections with “someone like me” [21,49]. However, while the power of peer support was well recognized among our participants, some expressed awareness of potential negative events that can occur in online peer support and warned that not everyone may have such favorable opinions or understand exactly what peer support is. It has been recognized that the term “peer support” has been inconsistently defined in research [16] and is often not colloquially used in patient communities. There is often a lack of clarity or misperceptions of what peer support involves that might serve as a barrier to engagement [61]. Indeed, evidence suggests that some people are deterred from online peer support as they believe that they will not fit in, peer support will be unable to support their well-being, their posts will remain unanswered, or that they will be exposed to incorrect or misleading information [16,59,62]. Given that perceived usefulness of an app can predict engagement, considering the end users' opinion and understanding of peer support may be an important barrier or facilitator to engagement [63]. It will therefore be important to provide clear information to users on what peer support is and how potential adverse effects will be mitigated on CommonGround. Future work should also evaluate participants’ views of peer support before and after engaging with online peer support to develop an understanding of the relationship between perceptions of peer support and engagement with interventions.

### Limitations

The design of the CommonGround platform and the findings from the formal usability testing (think-aloud exercises, interviews) should be considered within the context of our participant demographics. The majority identified as women, were daily internet users, and had access to the internet at home. Our sample did however have varying levels of prior experience engaging with online forums (30% [3/10] of participatory design panel lived experience experts and 42% [5/12] of usability testing participants had no prior experience of using online forums), representing a range of different baseline expectations of what online forums should be like. Nonetheless, as frequent internet users, they may perceive and prioritize issues differently than those who use the internet less often, particularly as they have greater experience to compare the prototype to. Although 96% of UK households have access to the internet [64], internet usage is lower among those in older adults (eg, 99.5% of those aged 25-34 years versus 85.5% aged 65-74 years using the internet in the last 3 months [65]). As living with (multiple) long-term conditions is more common among older people, who therefore represent a significant proportion of those that could benefit from CommonGround, future work should prioritize exploring the perspectives of this group and investigating the potential role of digital exclusion. By recruiting a larger, more diverse sample as part of a future feasibility randomized controlled trial, we will capture a broader range of experiences, usability concerns, and insights into barriers, facilitators, and intervention improvements. We will purposively interview participants based on various factors, including age and engagement with CommonGround, with topic guides including focus on exploring barriers, such as digital exclusion, in greater depth. Findings from this future work will ensure that CommonGround can be developed further to align with any specific needs identified, including those of older adults and those who may experience digital exclusion.

Our participatory design panel and subsequent RAG also represent a group of patients who are actively engaged in managing their physical-mental well-being, have an interest in peer support, and are frequently online. As such, their perspectives and associated intervention development are potentially rooted in positive experiences and opinions of peer support. Further exploration of the views of individuals who are new to peer support and/or less active in their self-management would provide broader insights into their psychosocial needs and expectations of how a peer support platform should function.

It should also be noted that no formal assessment of depressive symptoms was conducted among participants or RAG members; however, all involved self-identified as having lived experience relevant to preventing depression among people with long-term physical health conditions. While this broad, inclusive approach enabled a range of different perspectives to be incorporated throughout the coproduction process, we cannot conclude that our findings are specifically applicable to those living with subthreshold depression. Future work could explore capturing formal measures of mental health and well-being but should be mindful of excluding the voices of lived experience experts solely based on score cutoffs. Our future feasibility and acceptability trial will offer opportunity to explore the perceived acceptability, feasibility, and appropriateness of the CommonGround platform exclusively among a sample of those living with subthreshold depression.

Due to the COVID-19 pandemic, usability testing and subsequent RAG meetings for iterative development were conducted online via videoconferencing software. For participants unfamiliar with Microsoft Teams, this unfamiliarity may have contributed to nervousness or influenced how they interacted with the platform prototype during the think-aloud exercise. Resource restrictions also meant that usability sessions were conducted by members of the research team involved in prototype development. In future usability testing, involving an independent facilitator could enhance objectivity and encourage more candid feedback. However, to mitigate any bias as best as possible during the think-aloud exercises, the research team avoided prompting or guiding participants, allowing them to navigate and troubleshoot issues independently.

Finally, our findings may not be applicable beyond the cultural context of the United Kingdom. The willingness of people to discuss their mental and physical health with other people, particularly those who are not included in their existing social circles, likely varies cross-culturally due to different norms around expressing emotions and the role of family/community, and stigma.

### Conclusions

For interventions to be successfully integrated into the self-management routines of people with long-term physical health conditions, it is critical that they are developed in collaboration with individuals who have lived experience. By dedicating significant time and resources throughout the coproduction cycle, we have successfully created an online peer support platform with embedded psychoeducation that is designed to support the mental well-being of people living with long-term physical health conditions. Through an iterative process of usability testing and prototype development, we refined the platform to ensure that it aligns with the needs and preferences of the target audience.

The CommonGround platform is now ready to be further evaluated with continued input from our RAG during the delivery of a trial to explore the feasibility and acceptability of CommonGround. The findings from the next stages will be critical in informing the preparation of a future large-scale efficacy trial. If the intervention is found to be feasible, acceptable, and clinically effective at preventing the progression of subthreshold depressive symptoms to comorbid major depressive disorder, CommonGround may offer a low-cost, scalable solution to bridge the current gap in psychological support for people with long-term physical health conditions who are at risk of developing comorbid major depressive disorder.

## Data Availability

Data are available upon request to the corresponding author.

## Appendix

Multimedia Appendix 1 Consent forms.

Multimedia Appendix 2 Think-aloud activity tasks.

Multimedia Appendix 3 Topic guide for semistructured interviews.

Multimedia Appendix 4 The CommonGround platform manifesto written by our coinvestigator with lived experience.

Multimedia Appendix 5 The results tables of the content analysis of transcripts of the think-aloud task and semistructured interviews.

Multimedia Appendix 6 Screenshots of the final prototype of CommonGround.

## Abbreviations

KCL: King’s College London
PPI: patient and public involvement
RAG: research advisory group
