# Supplementary material for: Coproducing an Online Platform for People With Long-Term Physical Health Conditions: Development and Usability Study
Source: J Med Internet Res. 2026 Mar 24;28:e79666. doi: 10.2196/79666 (PMC13058536; doi:10.2196/79666)
Supplement: Multimedia Appendix 1 [file jmir_v28i1e79666_app1.docx]

Multimedia Appendix 1. Electronic consent forms.

**
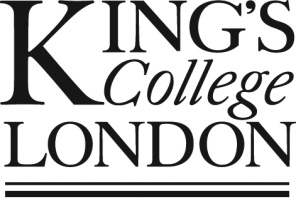
CONSENT FORM FOR PARTICIPANTS IN RESEARCH STUDIES**

**Please complete this form after you have read the Information Sheet and/or listened to an explanation about the research.**

**Title of Study: Online peer support for preventing comorbid depression in people with long-term conditions: focus groups**

**King’s College Research Ethics Committee Ref:________________**

Thank you for considering taking part in this research. The person organising the research must explain the project to you before you agree to take part. If you have any questions arising from the Information Sheet or explanation already given to you, please ask the researcher before you decide whether to join in. You will be given a copy of this Consent Form to keep and refer to at any time.

**I confirm that I understand that by ticking/initialling each box I am consenting to this element of the study. I understand that it will be assumed that unticked/initialled boxes mean that I DO NOT consent to that part of the study. I understand that by not giving consent for any one element I may be deemed ineligible for the study.**

1. I confirm that I have read and understood the information sheet for the above study. I have had the opportunity to consider the information and asked questions which have been answered to my satisfaction.
2. I consent voluntarily to be a participant in this study and understand that I can refuse to answer questions and I can withdraw from the study at any time, without having to give a reason. I understand that due to the nature of the focus group discussion it will not be possible to withdraw my data after taking part.

1. I consent to the processing of my personal information for the purposes explained to me in the Information Sheet. I understand that such information will be handled in accordance with the terms of the General Data Protection Regulation.
2. I understand that my information may be subject to review by responsible individuals from the College for monitoring and audit purposes.

5. I understand that confidentiality and anonymity will be maintained and it will not be possible to identify me in any research outputs.

6. I agree to be contacted in the future by King’s College London researchers who would like to invite me to participate in follow up studies to this project, or in future studies of a similar nature.

7. I understand that the information I have submitted will be published as a report and I wish to receive a copy of it.

8. I consent to the focus group being audio recorded, with possible use of anonymous quotations in reports and publications.

9. I agree to maintain the confidentiality of focus group discussions

**__________________ __________________ _________________**

**Name of Participant Date Signature**

**__________________ __________________ _________________**

**Name of Researcher Date Signature**


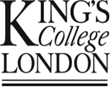


**CONSENT FORM FOR PARTICIPANTS IN RESEARCH STUDIES**

**Please complete this form after you have read the Information Sheet and/or listened to an explanation about the research.**

**Title of Study: Online peer support for preventing comorbid depression in people with long-term conditions: user testing**

**King’s College Research Ethics Committee Ref:________________**

Thank you for considering taking part in this research. The person organising the research must explain the project to you before you agree to take part. If you have any questions arising from the Information Sheet or explanation already given to you, please ask the researcher before you decide whether to join in. You will be given a copy of this Consent Form to keep and refer to at any time.

**I confirm that I understand that by ticking/initialling each box I am consenting to this element of the study. I understand that it will be assumed that unticked/initialled boxes mean that I DO NOT consent to that part of the study. I understand that by not giving consent for any one element I may be deemed ineligible for the study.**

1. I confirm that I have read and understood the information sheet dated for the above study. I have had the opportunity to consider the information and asked questions which have been answered to my satisfaction.

1. I consent voluntarily to be a participant in this study and understand that I can refuse to answer questions and I can withdraw from the study at any time, without having to give a reason. I understand that I can withdraw my data for up to weeks after taking part in the study.

1. I consent to the processing of my personal information for the purposes explained to me in the Information Sheet. I understand that such information will be handled in accordance with the terms of the General Data Protection Regulation.

1. I understand that my information may be subject to review by responsible individuals from the College for monitoring and audit purposes.

5. I understand that confidentiality and anonymity will be maintained, and it will not be possible to identify me in any research outputs.

6. I agree to be contacted in the future by King’s College London researchers who would like to invite me to participate in follow up studies to this project, or in future studies of a similar nature.

7. I understand that the information I have submitted will be published as a report and I wish to receive a copy of it.

8. I consent to my user-test/interview being audio recorded, with possible use of anonymous quotations in reports and publications.

**__________________               __________________              _________________**

**Name of Participant                 Date** **Signature**

**__________________               __________________              _________________**

**Name of Researcher                 Date** **Signature**
