# Supplementary material for: Coproducing an Online Platform for People With Long-Term Physical Health Conditions: Development and Usability Study
Source: J Med Internet Res. 2026 Mar 24;28:e79666. doi: 10.2196/79666 (PMC13058536; doi:10.2196/79666)
Supplement: Multimedia Appendix 2 [file jmir_v28i1e79666_app2.docx]

## Multimedia Appendix 2

Think-Aloud activity tasks.

| **Task #** | **Task description and prompt to stimulate verbalizations (if needed)** |
| --- | --- |
| Task 1 | Browse around for 1-2 min providing your feedback  Prompt: Talk us through what you are thinking. What is your first impression of this page? Who is the target audience? What do you think the purpose of the website is? |
| Task 2 | Create an account  Prompt: How easy was it to create an account? |
| Task 3 | Browse around the site for 1-2 minutes?  Prompt: Talk us through what you are thinking. What do you think the site does? |
| Task 4 | Find a resource and view it  Prompt: How easy was it to find the resource? |
| Task 5 | Add a social interaction and/or comment to the post  Prompt: Talk us through what you are thinking. What do the icons mean? What do you think of them? |
| Task 6 | Browse the resources section  Prompt: What do you think of the resources? |
| Task 7 | View posts from other community members  Prompt: How easy was it to find posts? |
| Task 8 | Post something to the community  Prompt: What did you think of the three post options? |
