# Supplementary material for: Coproducing an Online Platform for People With Long-Term Physical Health Conditions: Development and Usability Study
Source: J Med Internet Res. 2026 Mar 24;28:e79666. doi: 10.2196/79666 (PMC13058536; doi:10.2196/79666)
Supplement: Multimedia Appendix 3 [file jmir_v28i1e79666_app3.docx]

## Multimedia Appendix 3

Topic guide for semi-structured interview.

1. **What did you think about the platform you used here today?**

Prompts:

- *Positive experience*
- *Negative experience*
- *Anything that you liked?*
- *Anything that you disliked?*
- *From signing in through to completing each activity*

1. **Was it clear what the purpose of the platform was?**

Prompts:

- *Who it is for?*
- *What it is for? -* is there anything that would make you trust / mistrust this platform?

1. **How did you find navigating the platform and finding what you were looking for?**

Prompts:

- *Easy*
- *Difficult*
- *Why?*
- *Did you find anything confusing or frustrating?*

1. **Was there anything you looked for on the platform** **that you could not find?**

Prompts:

- *What additional features/functionality would you like to see?*
- *What additional content would you want?*

1. **How did you find the visual design and layout?**

Prompts:

- *What did you like the most?*
- *What did you like the least?*
- *What you would change?*
- *What would you keep?*
- *Thoughts on the name/ branding*

1. ***What positive and negative adjectives would you use to describe the site?***

Prompts:

- *What could be improved?*
- *What did you think of the terminology/tone?*
- *Experience with other platforms?*

1. ***How did you find the content and psychoeducational materials?***

Prompts:

- *Useful/ not*
- *Relevant/ not*
- *Accessible/ not*
- *Easy to understand/ difficult to understand*

1. ***The content you saw on the platform has not yet been finalised as we want to take on board as much feedback as possible from you. How can the content and psychoeducational materials be improved?***

Prompts:

- *How?*
- *Experience with other platforms?*
- *What would you trust, or not trust?*

1. **Where would you like to hear about a site like this?**

Prompts:

- *Via other support networks*
- *Social media*
- *Clinical care team*
- *G.P*
- *Testimonials from users*
- *What sources of advertising and information about this platform would increase your likelihood of using a platform like this?*
- *Where would you start to look for a resource like this?*

1. **What would you hope to gain from using the platform?**

Prompts:

*Support*

*Meeting others*

*Anything from a psychology perspective?*

- *Anything from a physical health perspective?*

1. **Why would you use a platform like this?**

Prompts:

- *If yes, why?*
- *If not, why?*

1. **Finally, do you have any further comments or suggestions about how the platform could be made more user-friendly?**

Prompts:

- *Other ideas*
- *Anything we have missed?*
- *Anything you strongly like or dislike?*
- *Is there anything else anyone would like to mention?*

*[end]*

***Probes used throughout as needed:***

- Would you give me an example of what you mean?
- Can you tell me more about that?
- Can you clarify that please?
