# Supplementary material for: Coproducing an Online Platform for People With Long-Term Physical Health Conditions: Development and Usability Study
Source: J Med Internet Res. 2026 Mar 24;28:e79666. doi: 10.2196/79666 (PMC13058536; doi:10.2196/79666)
Supplement: Multimedia Appendix 4 [file jmir_v28i1e79666_app4.docx]

## Multimedia Appendix 4

**The CommonGround platform manifesto written by our co-investigator with lived experience.**

Our diagnoses may be different,

but we are united in dealing with the challenge

when mind and body collide.

This is a two way street;

when we share we can learn from others

and see our experiences in a new light.

We are a community connected by health

There is no need to apologise for

what our bodies may do or not do.

What works for you may not work for me.

We are here to give and get support,

To learn, share, and grow.

This is a place to simply be present

And stand on CommonGround
