# Supplementary material for: Coproducing an Online Platform for People With Long-Term Physical Health Conditions: Development and Usability Study
Source: J Med Internet Res. 2026 Mar 24;28:e79666. doi: 10.2196/79666 (PMC13058536; doi:10.2196/79666)
Supplement: Multimedia Appendix 5 [file jmir_v28i1e79666_app5.docx]

## Multimedia Appendix 5

**Table S1.** The results of the content analysis of transcripts of the Think-Aloud task and semi-structured interviews.

| **Theme: Features and functions** | | | | | |
| --- | --- | --- | --- | --- | --- |
| **Codes and Code Frequency** | **Subcode** | **Description** | **Example quote(s)** | **Platform modifications and new development** |  |
| **Additional features** 75% of participants with 43 references | **About Us** | Participants expressed the need to know more about CommonGround as it is an unfamiliar and new website | *“So, if I was in a website, I would be looking, if it was somebody, I wasn’t familiar with, um, I would be looking for their about us, for them to tell me what they do.”* [P10] | **[1] Created an ‘About Us’ page** including the sections ‘meet the team’ and contact information.  **[2] Created a manifesto** that reflects the mission of CommonGround |  |
|  | **Search Bar** | The need to be able to easily search for what they are looking for | *“I would seriously just give me a search bar and let me write in what I want to look for.”* [P10]  *“I think if there’s, like, a search option, where you can, like, type in your own kind of, um, like you want to look up. Or like a filter to, um, find articles from what you want to search for your own condition kind of thing. Um, so you know, it’s easier to find what you’re looking for.”* [P6] | **[1] Created a search function** that filters relevant content via keywords or phrases.  [2] Ensured that search results can be **filtered by relevancy or recency**.  [3] Allowed searching by **free text** or by selecting the **content tags** users have assigned to their posts |  |
|  | **Moderation** | The need for features to report content, and a comprehensive moderation policy | *“Maybe also add, if you do break these rules, we’ll have to deactivate your account or something.”* [P4] | [1] Developed the **Moderation and Safeguarding Policies**  [2] Created webpages to display these policies  [3] Made the **‘flag this post’ feature** more eye-catching and added a field for the user to explain why they are flagging the post to facilitate the moderator’s decision making  **[4] Created notifications** to notify the user that a moderator has reviewed the post  [5] Used the term **‘flag’** rather than ‘report’. |  |
| **Content and Materials**  100% of participants with  73 references | **Positive views** | The variety of resource topics, formats (PDFs, videos), ease of accessing and downloading the resources was appreciated | *“To know that there are resources like that which I almost would have killed for, per se, at that time would, would mean that yes, this is why I would use this.”* [P1]  *"They were easy to open"* [P5] | [1] Ensured all the resources are **easy to open and download**  **[2] Gathered more resources** on topics relevant to people living with long-term conditions |  |
|  | **Frame and tone** | Negative perceptions of resources that had negative language and tone | *"Are you on the edge? Are you on the edge of what? Killing yourself? What is it, what is it with this negativity?”* [P10] | [1] Developed our **branding guidelines** including a brief on how CommonGround ‘speaks’.  [2] Created **site copy to introduce each resource** that matches this brief and clearly highlights what the resources is about and highlight key things of interest to encourage users to view the resource. |  |
|  |  |  |  |  |  |
|  | **Resource visuals** | Resource visuals were inconsistent with the platform and the aim of resources | *"I just think the black and white is just…" […] "Going against… […] What it’s kind of try to say” […] “and comparing to, you know, the, the actual website, it’s so colourful”* [P12] | [1] Selected resources with inviting layouts and visuals where possible (visuals were largely controlled by the resource owner). |  |
|  | **Sign-posting** | Participants advocated for the signposting to condition-specific resources | *"In my view, should be signposting people… […] because there’s no point in you reinventing wheels and developing, um, resources and information […] because all of those resources currently exist, and they’ve been developed by the disease-specific charities."* [P7] | [1] Focussed on **signposting** to existing resources rather than creating our own resources.  **[2] Created a repertoire of trusted, reputable condition-specific resources** (e.g., NHS, NICE) |  |
|  | **Trust** | KCL is a trusted organisation, but it was unclear who the ‘experts’ endorsing the resources were | *"That what’s endorsed by experts? The platform’s endorsed by experts or the content?"* [P2 ] | [1] Updated the **site copy** on the resources page with clear explanations of who the ‘experts’ are. |  |
|  |  |  |  |  |  |
|  |  |  |  |  |  |
| **Community page**  75% of participants with 26 references | **Positive views** | The three posting options (thought, question, knowledge) and post reactions (“I hear you”; “This helped me”) were considered unique and were well liked | *"(about posting options) I liked those categories. That’s something I’ve not seen ever before."* [P2]  *"(about reactions) I think these tabs down here are great. Um, the I hear you, this is helpful and comments. […] the I hear you and this is helpful, it’s quite nice to see that."* [P8] | [1] Created an **additional posting option** of ‘feeling’ for when users wish to simply post how they feel. |  |
|  | **Easy to use** | Participants quickly felt that it was easy to use the community page | *"I think it would be easy to post a question or make your own posts."* [P6] |  |  |
|  | **Add to My Garden' uncertainty** | Participants liked being able to save their favourite posts, however, the ‘save to My Garden’ button was confusing for some | *"I’m not sure what the Add to Garden means. I might be a bit dumb [laughing] if I don't know, but I’m not sure, um, about that, if I’m honest."* [P12] | [1] Re-labelled the ‘add to my garden’ button to ‘save to my Garden’.  [2] Removed the button icon  [3] Made the ‘save’ button reactive (colour changing) to indicate successful save/unsave |  |
| **My Garden**  92% of participants with 37 references | **Positive views** | Most considered My Garden to be useful for separating specific content of interest for later referral | *“That really appeals to me, because I can, I can, yeah, grow my own library, oh, that’s heaven [laughs].”* [P12]  *“It’s amazing that you’ve got that there because if there’s something you’ve seen and you know you want to go back to it, you’ve got that there.”* [P8] |  |  |
|  | **The ‘My Garden’ name** | Participants were divided in their opinions of the ‘My Garden’ name, with some indicating it was inappropriate | *“It’s not a nice place, when you’re living with mental health or… And a garden’s a nice place.”* [P5]  *"That garden thing, I think that’s a very quite cute idea because it’s, like, kind of, mental health is especially maintained to gardening sort of thing. It’s, like, highly associated with gardening so it’s quite calming."* [P8] | [1] Alternative names were considered. Decision to keep ‘My Garden’, but include site copy to explain the purpose of the page  [2] Developed ‘how to use My Garden page’ guide |  |
| **Joining form**  100% of participants with 62 references | **Expected for sign-up to websites** | The CommonGround sign-up fulfils participants expectations of registration forms | *"To be honest, it’s, it’s really straight forward and it kind of makes it easier because it’s, it goes along with what the majority of the registered, sort of, websites go for."* [P8] | **[1] Added labels** to identify required versus optional fields.  **[2] Updated password requirements** to match NHS guidelines, with colour changing criteria so users can check they have met all criteria (e.g., Contain at least two numbers)  [3] Added **‘hide/unhide’ password button** |  |
|  | **Burdensome sign-up fields** | Gender, ethnicity, and date of birth field formats were not user-friendly and frustrating to use | *"I think some people wouldn't really know the options for ethnicity. So, probably, um, like a drop-down kind of option menu should be available."* [P6] | [1] Implemented **user-friendly** **fields** for gender and ethnicity (drop-down) and date of birth (free text) |  |
|  |  |  |  |  |  |
|  | **What am I agreeing to?** | The guidelines are helpful, but adding a description of CommonGround would prevent them from seeming confrontational | *"The guidelines, I think were brilliant"* [P4]  *"It's almost like these are all our rules and regulations, and you have to agree to all of these rules and regulations before we will even tell you anything. That was, that put me on my guard, really."* [P10] | **[1] Updated site copy** introducing the community principles.  [2] Added links to **Moderation** and **Safeguarding** **Policies** in relevant places in statements the user must agree to |  |
|  | **Reason for joining** | The forced selection of "What brings you to CommonGround?" was perceived as confusing and overly restrictive | *"Um, I think another thing that might be nice here is if you could have more than just one option. Because I would probably suggest that I’d like to tick all three"* [P11] | [1] Removed the ‘what brings you to CommonGround’ page.  **[2] Updated the site copy** on the landing homepage where the different potential benefits of using CommonGround are presented, indicating that users get access to all aspects of the platform. |  |
|  |  |  |  |  |  |
|  | **Instructions** | Instructions during sign-up and profile creation should be more explicit to guide users effectively | *"I’m just trying to think, is that you have to say something that is, what, relevant about yourself? Or you could just say, you know, I'm, you know, I am here [laughs]. I am a person. I'm not quite sure when they say, when you say, tell us a little bit about yourself. I mean, in what context? I just want to find out what, in what context that is."* [P1] |  |  |
|  | **User control** | Participants valued control over their personal information, but clearer instructions are needed regarding anonymity and what to include or disclose | *"Ooh, that’s good that you can choose what to put in about yourself, yeah. Because I’m a bit… I don’t like everything about myself plastered all over the place, so that’s good that you can choose what to put in."* [P2] | [1] Created a **privacy policy** page that details how users’ data is stored and used.  **To sign-up page:**  [1] Added **clear** **statements** relating to the **anonymity** of the platform, and the requirement for usernames to be anonymous.  [2] Added **clear** **labels** of what fields are **required versus optional**.  [3] Added text to inform users what **personal information** (e.g., mobile number) would be used for (e.g., used for two-factor authentication only)  **To ‘my profile’ page:**  [1] Added the option to **edit** their **profile** at any time.  [2] Added **privacy** **toggles** users can choose whether their pronouns, gender, ‘my story’ or short biography are public on their profile. |  |
| **Theme: Usability** | | | | | |
| **Codes and Code Frequency** | **Subcode** | **Description** | **Example quote(s)** | **Potential platform modifications and new development** |  |
| **Platform navigation**  100% of participants with 118 references | **Positive views** | Most participants felt it was user-friendly, even if they are not technologically adept | *"It was easy to navigate, very easy to navigate."* [P5]  *"I mean, I, I wouldn’t say that I’m fantastic in technology. So, it was… It’s, it’s… I think it’s quite technically friendly, and quite easy to access, to be honest"* [P9] | When adding **new** **features** **and** **functions**, ensured that they were **built in** **an intuitive way**.  For instance:  (1) Re-labelled buttons so that their function is clear (2) Clicking on own profile icon redirects to own profile page as expected |  |
|  | **Intuitive** | Navigation is easy when it aligns with participants' intuitive expectations, based on their experience with other websites | *"I think I’ve got so used to seeing a menu thing at the top that when I go on to any website, that’s the first thing I look for, is that menu."* [P10] |  |  |
|  | **Navigation bar** | Navigation bar is invaluable, but some did not initially notice the navigation bar | *“I think, uh, maybe it’s to do with these tabs up here, because I didn’t initially notice that they were there at all." […] "Um, I don't know whether, um, these may be bigger or, um… I’m trying to think if, if… I don't know. I just… I think, because I didn’t initially notice they were there."* [P12] | [1] Made text of the **navigation tabs larger**  [2] Made the **navigation bar interactive**, where the tab changes colour and is underlined when the mouse hovers over it  [3] Added a **‘are you in crisis’ banner** in contrasting colour above the navigation bar that redirects to the further support page  **[4] Added a bottom navigation bar** with tabs typically located at the bottom of the page, including ‘contact us’ and the privacy policy |  |
|  | **Where is the search bar?** | Participants suggested that any difficulties in navigation would be overcome by using search bar | *"And the search, because I'm really bad at navigating. Like, I tend to put something in the search bit to find it for me quickly."* [P10] | [1] As above, made improvements to the existing navigation bar, and added **two additional navigation bars** to improve accessibility of different webpages within the platform  [2] Created a **search** **function** that filters relevant content for keywords or phrases.  [3] Ensured that search results can be filtered by relevancy or recency.  [4] Allowed searching by free text or by selecting the content tags users have assigned to their posts |  |
| **Visual design**  100% of participants with 75 references | **Button icons** | Icons for the posting buttons were confusing | *“The things you have, the images that you have above the post a thought, share knowledge, ask a question, I was just wondering what that is, what those images depict”* [P1] | [1] Created **new icons that represented the function of the button**. For example, a thought bubble to accompany the create a thought button. |  |
|  |  |  |  |  |  |
|  | **Background visuals** | Background visuals and icon shapes were appreciated | *"I do like these, these, um, images and the squiggles. I don't know why I find them attractive. I just find them attractive."* [P10] | [1] Reviewed the **colour** **palette** of CommonGround to include muted (non-neon) tones. |  |
| **Visual layout**  92% of participants with  61 references | **Positive views** | Simple, clear and easy-to-process layout | *"I like the, I like the layout. Um, [mouth click], um, it’s quite clear to, sort of, see individual posts and it seems like a nice size to be able to digest."* [P11] | [1] Updated the **layout** in the **mobile** **version**, including adding white space between posts,  [2] Created **post previews** for longer posts, where users can click on the title to view the full post in article view.  [3] Created **continuous load**, so as users scroll to the bottom of the community feed, additional posts load. |  |
|  | **Uniformity** | Consistent layout throughout website pleases users | *"Just that continuity just throughout the whole platform”* [P12]  *"I like that that’s in the same format as what I’ve just been in in the community.”* [P2] |  |  |
|  | **Errors** | Homepage: top of the text is cut off | *"I'm actually missing the top of one of the pages."* [P1] | **[1] Resolved layout glitches** where top section of page is cut off |  |
| **Tone and Language**  100% of participants with 77 references | **Explicit, clear language** | Participants want explicit, clear, and accessible language throughout the platform | *"I’m a little bit confused on when you say active, is that more like exercising keeping active sort of thing or…?"* [P8] | [1] Developed our branding guidelines including a brief on how CommonGround ‘speaks’.  [2] Created **site** **copy** to introduce each resource that matches this brief and clearly highlights what the resources is about and highlight key things of interest to encourage users to view the resource. |  |
|  |  |  | *"you look at research that has really kind of quite difficult, um, linguistic, medical kind of language, and you, you want to try to get to a place whereby it, it’s more lay, type of thing, so that you can understand it."* [P1] |  |  |
|  | **Definitions** | Participants' knowledge of certain terms cannot be assumed, so providing definitions is necessary | *"some people might not know what mindfulness is"* [P5]  *"peer to peer" […] "I think at times, it could be slightly wordy." […] "It’s a term that I've come across, yes definitely. I'm not sure it’s term that everybody will know, though"* [P1] | [1] Where needed, **definitions** (approved by the RAG) were added to site copy.  [2] Included **resource(s)** on what **peer support** is**.** |  |
|  | **Garden-related terms** | Garden-related terms are too abstract and cause confusion | *"I don't like the word cultivate. It’s not, it doesn't mean anything to me other than, yeah, we grow. And in this context, that is not helpful."* [P10] | **[1] Reviewed garden-related terminology**, replacing terms to better reflect what users can do in My Garden (i.e., save posts) |  |
|  | **Function button labels** | The labels of different buttons need to clearly reflect the button function so users can quickly understand what the button does | *"if it had said post now or send now or something, that would have made me know that I wanted to do it."* [P10]  *"Be first. Three ideas. Be first. Be first to what? Be first there and be first there."* [P2] | [1] Reviewed **button** **labels** to ensure labels are clearly communicating button functionality |  |
|  | **Tone of the resources** | The tone is crucial for participant engagement; if a resource is perceived as negative, participants are less likely to use it | *"Are you on the edge? That, that sounds awful. Um, maybe something, do you need help? But not are you on the edge."* [P5] | [1] Created **site copy** of resource titles and introductions to each resource that matches how CommonGround ‘speaks’ and clearly highlights what the resources is about |  |
|  | **Context is important** | Context is essential for helping participants understand and engage with the information | *"Um, you say end, endorsed by experts…" […] "But who, who exactly?"* [P2]  *"Then it goes down to guiding principles. Once I saw that, I took it as being, these are our rules and you best not break it. So, it wasn't, to me, it didn't come across as having a friendly, warm, welcoming approach."* [P10] | [1] Created **site copy** to introduce different webpages, outlining the purpose of the page.  [2] Created an **‘About Us’ page** including the sections such as ‘meet the team’ and contact information. |  |
|  |  |  |  |  |  |
|  |  |  |  |  |  |
|  |  |  |  |  |  |
|  |  |  |  |  |  |
|  |  |  |  |  |  |
|  |  |  |  |  |  |
| **Theme: Platform branding** | | | | | |
| **Codes and Code Frequency** | **Subcode** | **Description** | **Example quote(s)** | **Potential platform modifications and new development** |  |
| **Platform branding**  67% of participants with 20 references | **Brand awareness** | The balance between beneficial brand awareness and over-commercialisation was acknowledged; participants want to know who is behind CommonGround without the branding being overwhelming | *“Honestly, no, it's not. But then I don't know if that's such a bad thing. Um, because it's… You're not trying to get your name and the branding across. It seems like you're trying to make the issue a more important thing, which is trying to help people with their mental health.”* [P8] | **[1] Refined the branding** of CommonGround, building branding guidelines including ‘how we speak’, our ethos, the manifesto, updated colour schemes |  |
|  | **Trust** | Trust and credibility stem from the KCL affiliation, but this affiliation could be more prominent | *"you’re under King’s College so that gives you that credibility anyway."* [P11]  *“if you just Google on the internet. If CommonGround came up, I might look, but I might think, well, well, who’s, who’s set this up exactly?”* [P2] | [1] Ensured **KCL** **logo** is added in prominent locations on the CommonGround platform, participant information, affiliated communications (e.g., blogs, participant emails) and recruitment materials.  [2] Adding BitJam (software developer) logos to relevant sections on the platform so that it is clear who we collaborate with |  |
|  | **Logo** | Logo does not communicate health or wellbeing or long-term physical health conditions | *“The branding isn’t completely clear in all honesty. I know CommonGround is probably the main logo. Um, reading Common Ground for the first time you wouldn’t exactly think it’s, like, health-related or anything”* [P6] | [1] To supplement the original logo, additional graphics in line with brand guidelines have been added |  |
| **Platform name**  83% of participants with  16 references | **Communicates a common space** | The name clearly communicates finding common experiences between users | *"That was a common ground. So, it was finding something, um… I liked that. Something common between people."* [P3] | [1] Review **site copy** of the landing homepage and developed the manifesto and ‘about us’ page that all communicate the purpose of the platform, the ethos, and how the community will be run. |  |
|  | **Link with mental health** | The name has limited association with mental health or wellbeing | *"I do think that’s a really good name, but, um, I don’t know whether it says enough. " […] "It depends how people are introduced to Common Ground." […] "If it’s something you find, you know, if you Google, if you Googled psychological services, and Common Ground came up, then I don’t think it would really matter."* [P8] | [1] At these stages of the research, visibility in search engines is not a priority as the platform is closed to research participants only. All potential research participants will be introduced to the platform as a mental health intervention via the recruitment materials. |  |
| **Advertising**  100% of participants with  28 references | **Routine locations** | CommonGround needs to be advertised to people at locations (physical or digital) that someone living with a long-term condition would frequently encounter (e.g., GP surgery) | *"To be able to reach people, you need to, you know, have it in, you know, more places or where people naturally would go to."* [P12]  *"as well as, like, GPs, um, physiotherapy. Um, other sites? I guess, um, GP websites as well, um, even a kidney research websites and charity websites as well as their own hospital websites"* [P6] | **[1] Developed pathways for advertising** the CommonGround along the routes that patients naturally encounter in their routine care and daily lives. This includes via GP and routine outpatient clinics, via long-term condition charities.  [2] Developed various different **recruitment** **materials** that can suit a range of needs, from physical information sheets that can be taken home, to QR codes linking to the virtual participant information, and social media images. |  |
|  | **Trusted sources** | Advertisements must come from a trusted source for the platform to be considered credible | *"I think it carry, it would carry more weight. You know, if a, if a professional has recommended something"* [P2]  *"If it’s (CommonGround) not endorsed by someone who you trust, um, and that they can actually say that they believe in it, it could be, it could be misinformation."* [P9] | **[1] Developed pathways for advertising** the CommonGround along the routes that patients naturally encounter in their routine care and daily lives. This includes via GP and routine outpatient clinics with Guys and St Thomas’ and Kings College Hospitals. |  |
|  | **What can I gain?** | Advertisements must be inviting and present potential gains from using the platform | *"Do you think you can benefit from blardy blah, you know, this platform or that platform? Or we have a platform or we have a project or something like that, you know, which is codesigned by people with long-term health conditions in partnership with, you know, other medical professionals here, kind of thing"* [P1]  *"they need to be convinced that a platform is a good place to turn to"* [P9] | [1] Developed various different **recruitment** **materials** that can suit a range of needs, from physical information sheets that can be taken home, to QR codes linking to the virtual participant information, and social media images. In these materials, the potential for social support from people ‘like me’ was emphasised. The recruitment materials also **highlight that the platform has been co-produced** by people with lived experience, and also highlights the unique ‘all diagnoses welcome’ approach. |  |
| **Theme: Barriers and facilitators** | | | | | |
| **Codes and Code Frequency** | **Subcode** | **Description** | **Example quote(s)** | **Potential platform modifications and new development** |  |
| **Barriers to access**  83% of participants with  31 references | **Suitability** | People might not engage with the peer support platform if they do not believe that the platform is suitable for themselves | *"they might also be averse to going somewhere and talking about themselves, because they’ve never wanted to talk about themselves."* [P9]  *"it comes back to the, the generalist versus the targeted and specific…" […] "Um, you know, I think that most people will, will err towards the specific because they’re going to find lots of other people like them."* [P7] | **[1] Included resource(s) on peer support** to help people understand what peer support is, how it might suit them, and the potential gains from peer support.  [2] In our recruitment materials and platform branding, included our **‘all diagnoses welcome’** approach and rationale.  [3] Including **signposting** to other communities or resources in our recruitment materials for those who do not wish to engage in an ‘all diagnosis welcome’ approach |  |
|  | **What is peer support?** | Misconceptions/negative perceptions about peer support or not knowing about what peer support is | *“you’re on this platform practically every day. That’s not healthy”* [P7]  *"*[about peer support] *It’s a term that I've come across, yes definitely. I'm not sure it’s term that everybody will know, though."* [P1] | **[1] Included resource(s) on** **peer** **support** to help people understand what peer support is, how it might suit them, and the potential gains from peer support. |  |
|  | **Safety** | Concerns about the safety and appropriateness of content posted by other users of the site, including misinformation or promoting false treatments/products and how the platform would be moderated | *“It's just you know how there's like a lot of spam, a lot of scam, especially when it comes to links sort of thing. And you never know what you're clicking on. So, somebody who's got good intention and coming on the website, you don't know if that's going to be everybody's intention."* [P8] | [1] Added **two-factor authentication to** the login process to minimise risk of compromised accounts that might spread misinformation or malicious links  **[2] Moderation policy** with clear guidance on what can and cannot be shared/spoken about on the platform, with a moderation team who will enforce the policy  [3] A function that requires any **post with an attachment to be reviewed** by the moderation team ahead of going ‘live’ to ensure safety of attachments.  [4] Created our policy that although users are **anonymous** to one another, the research team are aware of the personal details linked to each out  **[5] New registered accounts must be approved** by the research team through the admin panel before they are granted access  [6] Users **cannot edit their own posts and requests to have their post deleted are reviewed** by the moderation team, which increases user accountability for what is shared. |  |
|  | **Accessibility** | If the platform cannot meet an individual’s accessibility and additional needs | *"maybe for people who are dyslexic, it might be harder for them, but. Um, so maybe use simpler words"* [P4]  "*I was just thinking about visual impairment." […] "Um, whether an option could be built in where it’s, uh, I guess, being read out to them or something instead."* [P12] | [1] Review of site copy to ensure simple, clear language is used throughout, and RAG-approved definitions are used when relevant.  [2] The platform is screen-reader compatible, with a section on the ‘about us’ page with instructions on using CommonGround with a screen reader |  |
| **Facilitators to access**  100% of participants with  41 references | **Trust** | Knowledge that CommonGround is co-produced with KCL, a trusted organisation | *“knowing that […] [it’s] codesigned by people with long-term physical health conditions." [Interviewer: Yeah. So, for you, would it be important that that’s really clear?] "Very important […] I mean, that should take, you know, being explained on, you know, the first page of any kind of document that, that, that is put out."* [P1]  *“because it’s endorsed by King’s College, it would give me the, um, the opinion, and the impression that it was, um, it was fairly accurate and secure."* [P9] | [1] Ensured **KCL** **logo** is added in prominent locations on the CommonGround platform, participant information, affiliated communications (e.g., blogs, participant emails) and recruitment materials  [2] The **recruitment** **materials** also highlight that the platform has been co-produced by people with lived experience, and also highlights the unique ‘all diagnoses welcome’ approach. |  |
|  | **Awareness through recommendations** | Recommendation from a trusted source that CommonGround could ‘fulfil my needs’ | *“Um, if you searched it just by yourself, you would be always concerned that whether it’s a legit site, or whatever. So, I think it really needs to come from the health authority, um, from, from the councils. And from the people that, you know, that people trust, the GPs and everything."* [P9] | **[1] Developed pathways for advertising** the CommonGround along the routes that patients naturally encounter in their routine care and daily lives. This includes via GP and routine outpatient clinics with Guys and St Thomas’ and Kings College Hospitals. |  |
|  | **Flexibility** | Flexibility to use and engage with the platform as the user chooses | *"just that it felt like, [mouth click], you’ve got, you’re in control of what you put in there and what you don’t."* [P11]  *"You don’t have to constantly look at it. So, I think that’s maybe the thing. Where you can dip in and out"* [P3] | [1] Developed **a further support and crisis page** and **moderation** **policy** which includes information on when it might be useful to take a break and using the platform safely. |  |
| **Confidence with technology**   67% of participants with 20 references | **Suitable for all** | CommonGround was considered as suitable for all levels of technology competency | *"somebody who’s not very tech-savvy to somebody who can code or whatever. It just seems like it’s very open to a lot of people”* [P8]  *"I mean, I, I wouldn’t say that I’m fantastic in technology. So, it was… It’s, it’s… I think it’s quite technically friendly, and quite easy to access, to be honest."* [P9] | |  |
|  | **Low-skilled users** | Participants who considered themselves as low skilled felt confident to use the platform, often acknowledging that they might take longer to familiarise with the platform. Only one participant expressed their technological abilities was associated with anxiety | *"And I don't mind admitting that sometimes I'm slow, where that's concerned, because I just, you know, I think I sometimes… I get there in the end, but I get there slower."* [P1]  *"I'll get a bit anxious on it. Because it's like, it's, it's technology."* [P10] | |  |

**Table S2.** Insights and example quote for the theme ‘ethos and values’ from the UT: Stage one.

| **Theme: Ethos and values** | | | |
| --- | --- | --- | --- |
| **Codes and Code Frequency** | **Subcode** | **Description** | **Example quote(s)** |
| **Platform purpose**  100% of participants with 81 references | **Purpose identified** | All participants commented on the platform's purpose, with most highlighting its dual focus on physical and mental health, as well as the peer support and psychoeducation elements | *"it’s a bit of a double-whammy then, isn’t it?" […] "You, kind of, get the support from, [tinkle], from peers as, as well as, um, professional, kind of, advice. Even if it’s not advice, it’s, er, it’s a step in the right direction of, [rattling], if you’ve got this try that."*  [P11]  *"I mean, as well as your physical health conditions, I think it would help tremendously with my mental health"* [P6] |
|  | **Clear purpose** | Some participants explicitly stated that the purpose was clear | *"It is what it says on the tin, as it were. You, you can… You can post. You can share. You can share your experience and what-have-you."* [P7]  *"It was quite calming, it was quite straight forward, it was clear. You could kind of see what it is. It’s not going and being like, oh what is this for sort of thing?"* [P8] |
|  | **Broad suitability** | Some participants shared that the platform was suitable for those living with *any* type of long-term condition | *"I know it's not restricted to, like, kidney disease, for example. So, you can read about, um, other conditions, other, um, kind of problems other people go through. So, you can expand your knowledge through this website as well and find things out about, um, even your own illness, for example, and what other people are going through, their treatments. I think it will definitely help people to increase their knowledge and learn more about that condition as well as other conditions."* [P6] |
| **Condition specific requirements** 25% of participants with 11 references | **Symptom tracking** | A record keeper: a place to record symptoms, feelings etc | *"Okay, so you have something that’s called My Garden." […] ". Obviously, it's a library. But which I think is actually quite helpful because this, this is, in a way, this is, er, like a kin to me. When I was first diagnosed about keeping a diary almost, about things that happened to me and how I was feeling generally, as well, you know, as well as just writing down that I needed to take this medication and that medication at this and that time. So, it was that kind of, er, thing that I used to do, keep a diary." [...] "So, I kind of wrote down, you know, how I was feeling, how the drugs that I were taking, what kind of impact they were having on me, etc, etc. What kind of, and you know, how much pain I was in on certain days kind of thing, and how long did it last."* [P1] |
|  | **Cognitive overload** | Simple layout with small chunks of text is suitable for those whose conditions have cognitive difficulties | *"It doesn’t overload your mind. Because, um, I know a lot of, I mean, obviously, I don’t have other conditions that other pe, other people who use this will do, but particularly for MS, one of, one of the difficulties is cognitive, cognitively." […] "And cognitively, um, I find things quite difficult, like lots of information. I can read it all, no problem, and I can understand it whilst I’m reading it." [...] "So, the fact that I can navigate so easily, and there’s only four things there. So, even if I couldn’t remember which category something came, came under, I could just simply click on them all and find it, it’s no big deal."* [P2] |
| **Peer support experiences**  83% of patients with 31 references | **Previous experiences** | Some participants spoke about formal peer support experiences, involving organised groups, and others about informal peer support that had developed organically | *"It was the first time that I was, you know, in the presence of like, like-minded people with similar conditions, for us to be sharing our stories." [P1]*  *“we formed this like, it was, it was, it was almost like a support group." […] "And it was all just lovely because these were people who knew what you were going through at three o'clock in the morning when you can't bloody get enough air in. And you don't want to disturb your partner. Are you with me? They knew what that felt like. Um, and we could, so we supported each other in those terms."* [P10] |
|  | **Positive views** | Many participants shared positive views of peer support | *"And so talking to somebody or speaking with somebody or reading something that somebody else has maybe been dealing with longer or has a different way of dealing with it it's good, it's good to, kind of, get their experiences." [P8]*  *""it’s a way of keeping connected. And that connection is so important."* [P3] |
|  | **Negative views** | A few patients spoke about negative views of peer support | *"i’m always astonished at the number of people in [existing peer support platform] that go on there on a very regular basis, and I kind of feel, you know, you’re on this platform practically every day. That’s not healthy [laughs], in my view. Do you know what I mean? I think people should be, you know, getting on with their lives, um, and maybe it says something about people who use platforms on a regular basis, even when they’re well-controlled."* [P7] |
| **Platform importance**  92% of patients with 41 references | **The gap is identified** | The need for the platform was well recognised, with suggestions that CommonGround is addressing a gap in healthcare | *“when I saw something like this, because of, you know, what I’ve been involved in before, is ‘much needed’"* [P1]  *"there's nothing really else like this at the moment that I know of. Um, so I would definitely use something like this."* [P6] |
|  | **Everything together** | Participants appreciated how CommonGround brings together reliable resources and community support in one place | *"I think a resource like this is going to come into its own because that is what people are looking for. Getting that advice, getting that peer support, that whatever, you know, medical support, all in one place."* [P2] |
|  | **Holistic** | The platforms holistic perspective that combines mental and physical health is critical, as this is the only appropriate way to capture the experiences of those living with long-term conditions | *"it’s, [laughing], a combination of the two, not just pigeon-holed into one or the other." [P11]*  *[in reference to mental health needs/wellbeing alongside the long-term condition] "I think we've been waiting probably for a long time for things like this to, to come out and take, kind of, you know, to take consideration of, of that, that need, those needs as well."* [P1] |
| **Meaning of co-production**  50% of patients with 13 references | **Essential** | Some participants expressed that co-production should be essential when developing interventions | *“Co-design is essential if you’re doing anything for a, a particular audience. You, you, you must include representatives of that audience if you want to get a, a, a fully rounded, um, end product"* [P7]  *"it's important that people with long-term health conditions are, you know, have an investment or say in it. That's what I think is important. Because I think a lot of these things are designed for, you know, with, to do to, to people, as, as opposed to with people."* [P1] |
|  | **Both are required** | To produce a high-quality intervention, input from *both* patients *and* experts or academics are essential | *"We’ll always include both because you need… Um, especially when you’re dealing with complex conditions, you need the, um… The rigour, if you like, of having, um, that evidence base"* [P7] |
|  | **Patients are invaluable** | Patients provide invaluable insights based on their lived experience | *"You know, it’s just like a duh moment. Why wouldn’t we be experienced in that kind of thing?"* [P1]  *"We may not be able to communicate it as well as, you know, somebody with a PhD or, you know, a big title before their names. But certainly, if you know what I’m saying, we, you know, we know what we live and we live what we know."* [P7] |
| **Power of peer support**  67% of patients with 22 references | **Reciprocal** | The reciprocal nature of the community is important | *"Knowing that there’s somebody else that you can just chat to or can understand. And it’s also nice to be able to give back as well when you’re feeling that you’re able to do things"* [P11] |
|  | **Detachment from social circles** | Peer support can be powerful because it is detached from their immediate social circles | *"a lot of people I, I, I would imagine would, would talk to somebody on the bus or whatever, before they’d talk to family, or friends even. […] The good thing about that is nobody knows who I am."* [P5] |
|  | **Alleviating loneliness** | Peer support can be particularly powerful when experiencing loneliness and the 'why me' feelings during the diagnosis process | *"I think it's, er, you know, it is, being diagnosed with any long-term condition as well, I think is, I think I remember saying to you, when I was first diagnosed, and how my, my life had been changed, kind of thing, you know? You think, at that time, you think, that you're the only one almost that has this, you know? And it’s silly maybe to think that because you know, you know, anybody with a, with a, with a disease or whatever, there's probably going to be, you know, umpteen other people that's going to have it. But you don't think that way."* [P1] |
|  | **A potential negative reminder** | Acknowledgement that peer support might always be a positive experience | *"I think that most people would prefer not to have to be using forums and apps and constantly reminding themselves that they’ve got a long-term condition. Um, and, you know, do I really want to wake up every morning and turn to my app and tell it how I’m feeling today or tell it what my pain level is today? I’d rather just get up and get on with my life."* [P7] |
