# Supplementary material for: Coproducing an Online Platform for People With Long-Term Physical Health Conditions: Development and Usability Study
Source: J Med Internet Res. 2026 Mar 24;28:e79666. doi: 10.2196/79666 (PMC13058536; doi:10.2196/79666)

## Multimedia Appendix 6

**Screenshots of the final prototype of CommonGround**

Screenshot 1. The landing page of CommonGround


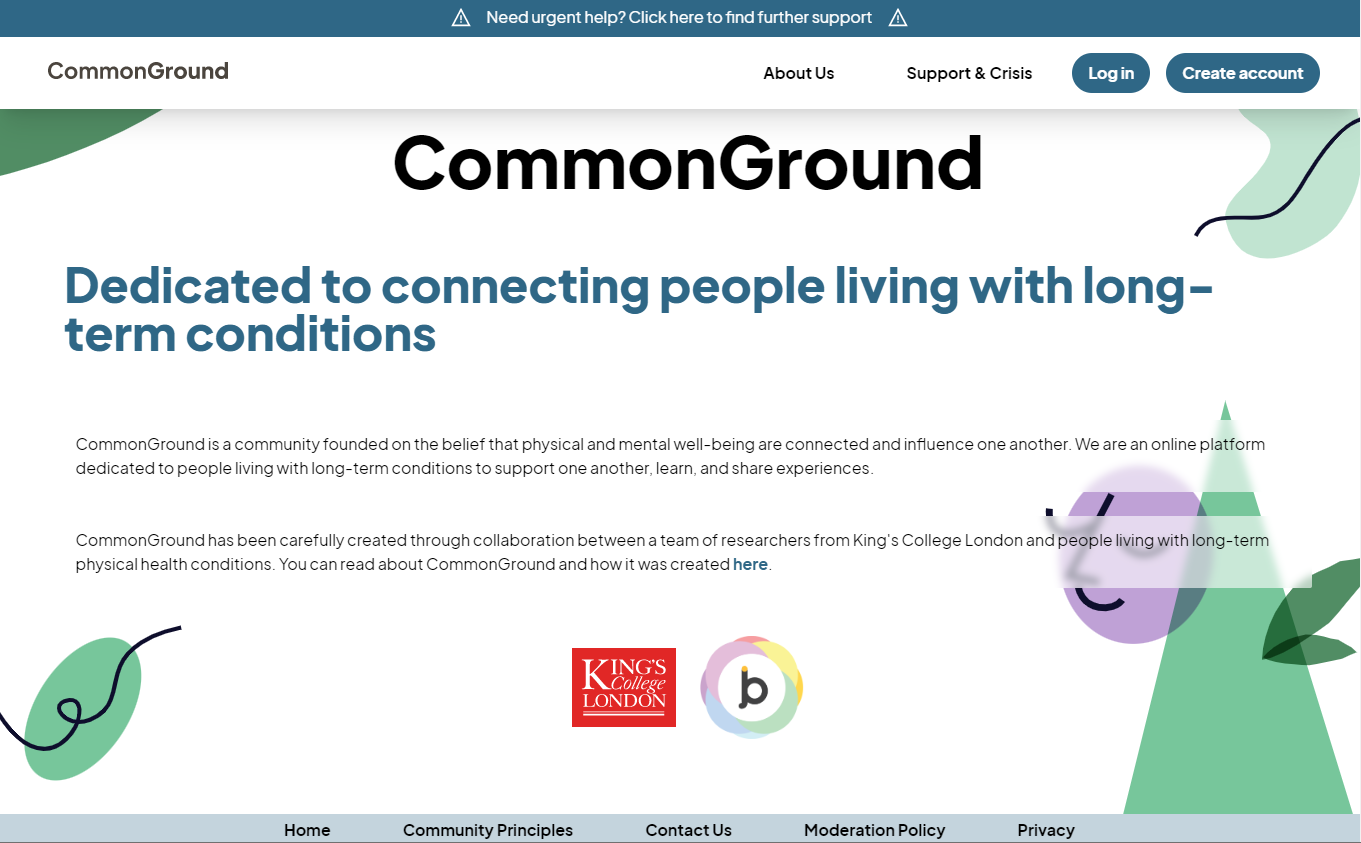


Screenshot 2. The community feed of CommonGround (peer support component)


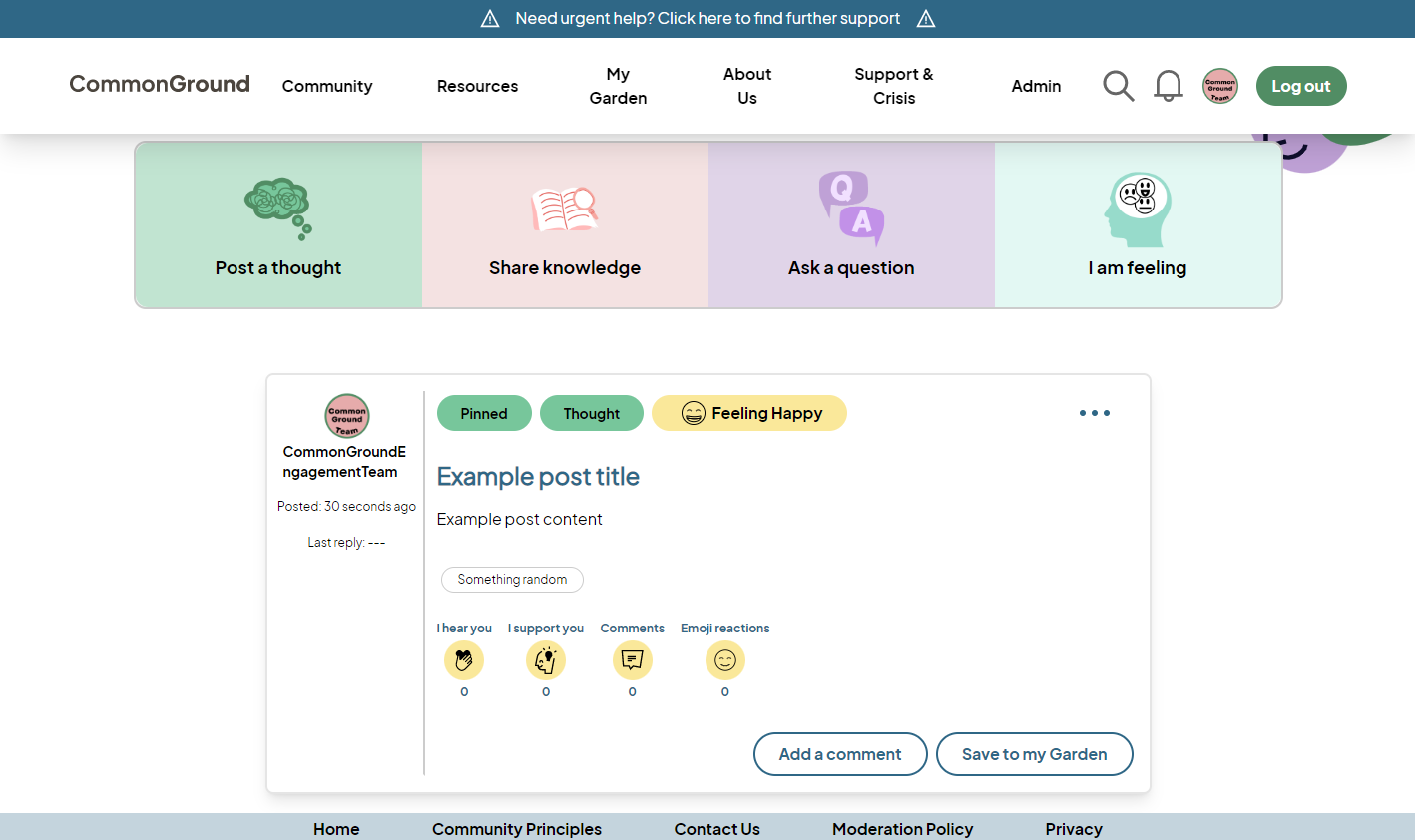


Screenshot 3. The resources page of CommonGround (psychoeducation component)


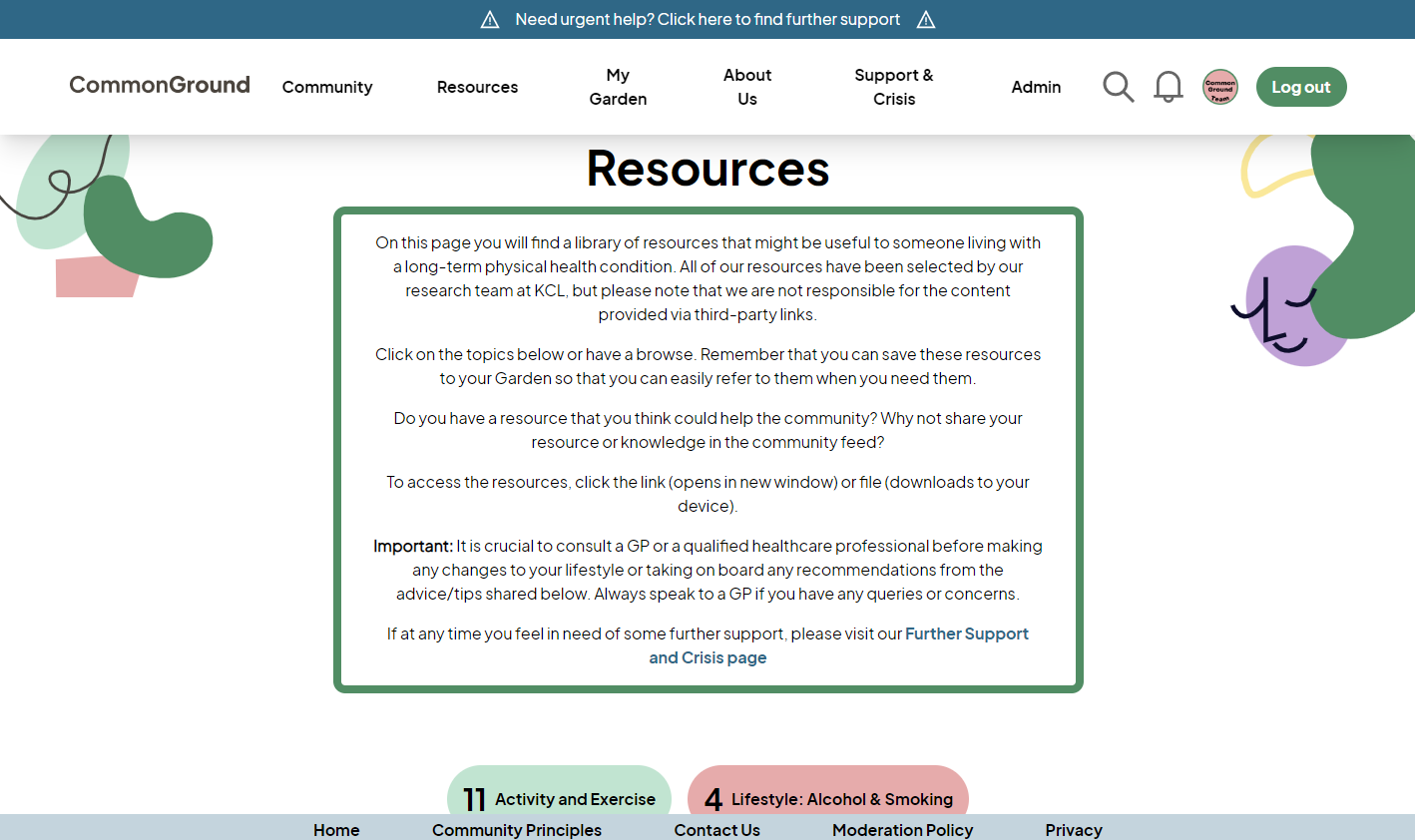


Screenshot 4. The ‘My Garden’ page of CommonGround


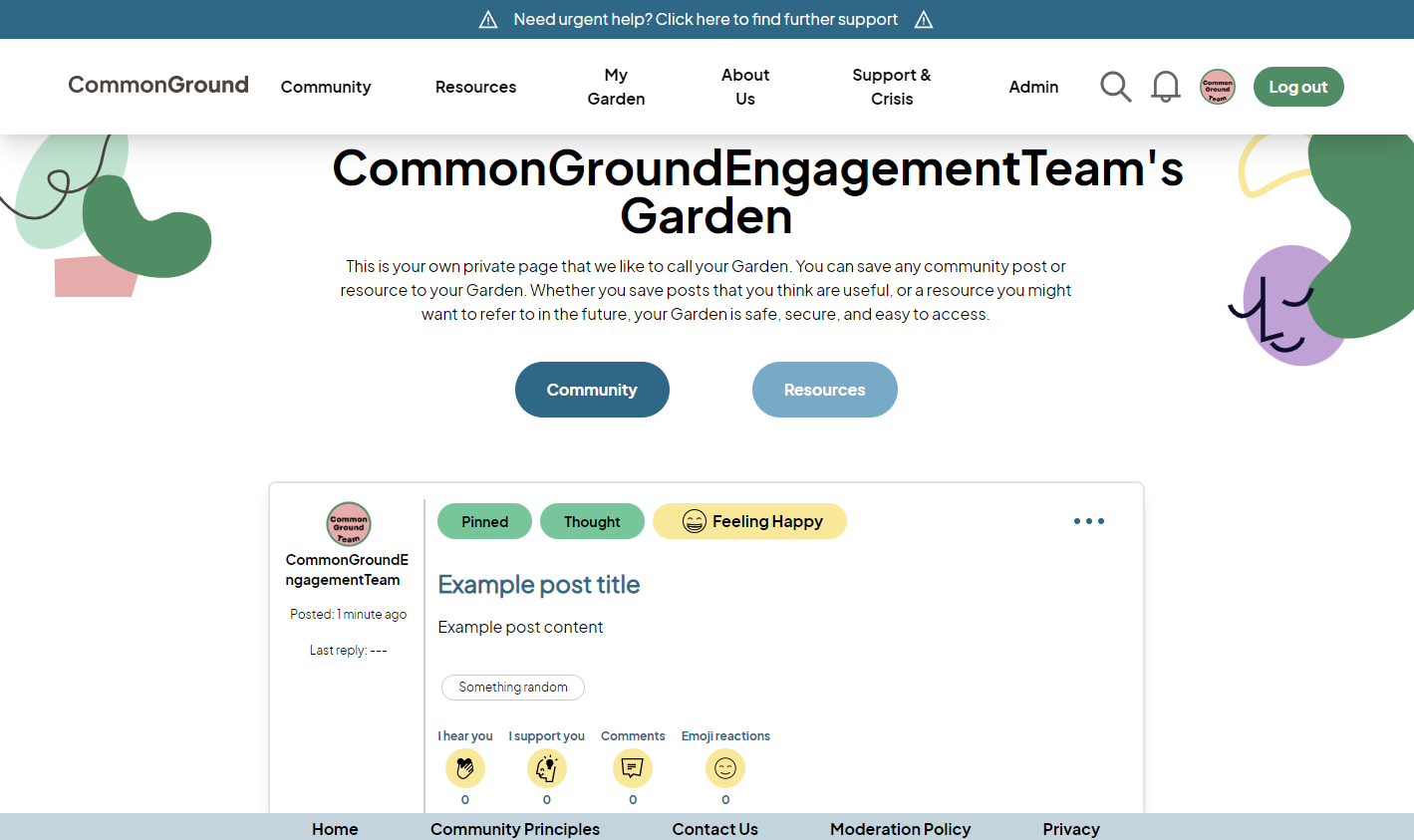


Screenshot 5. Search box and search results page of CommonGround


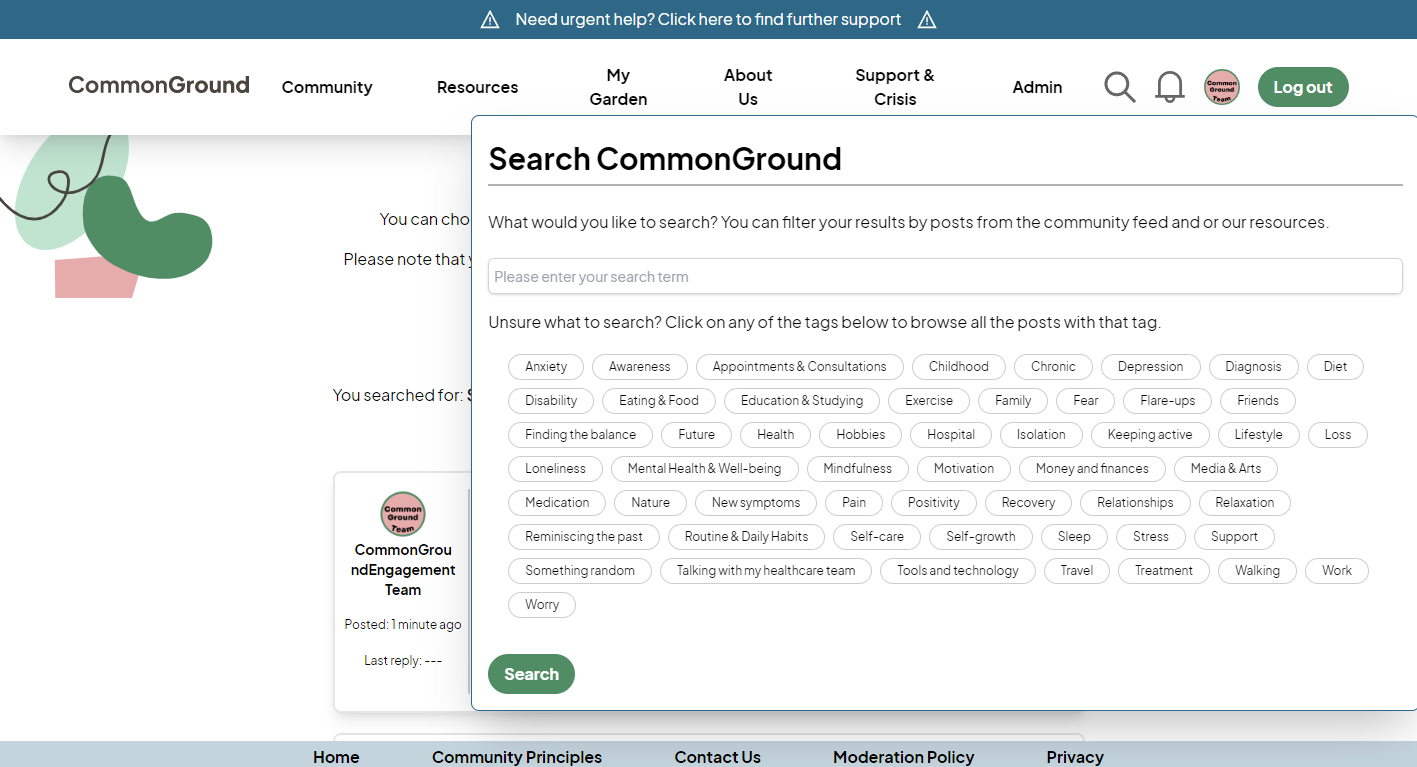


Screenshot 6. Further support and crisis page


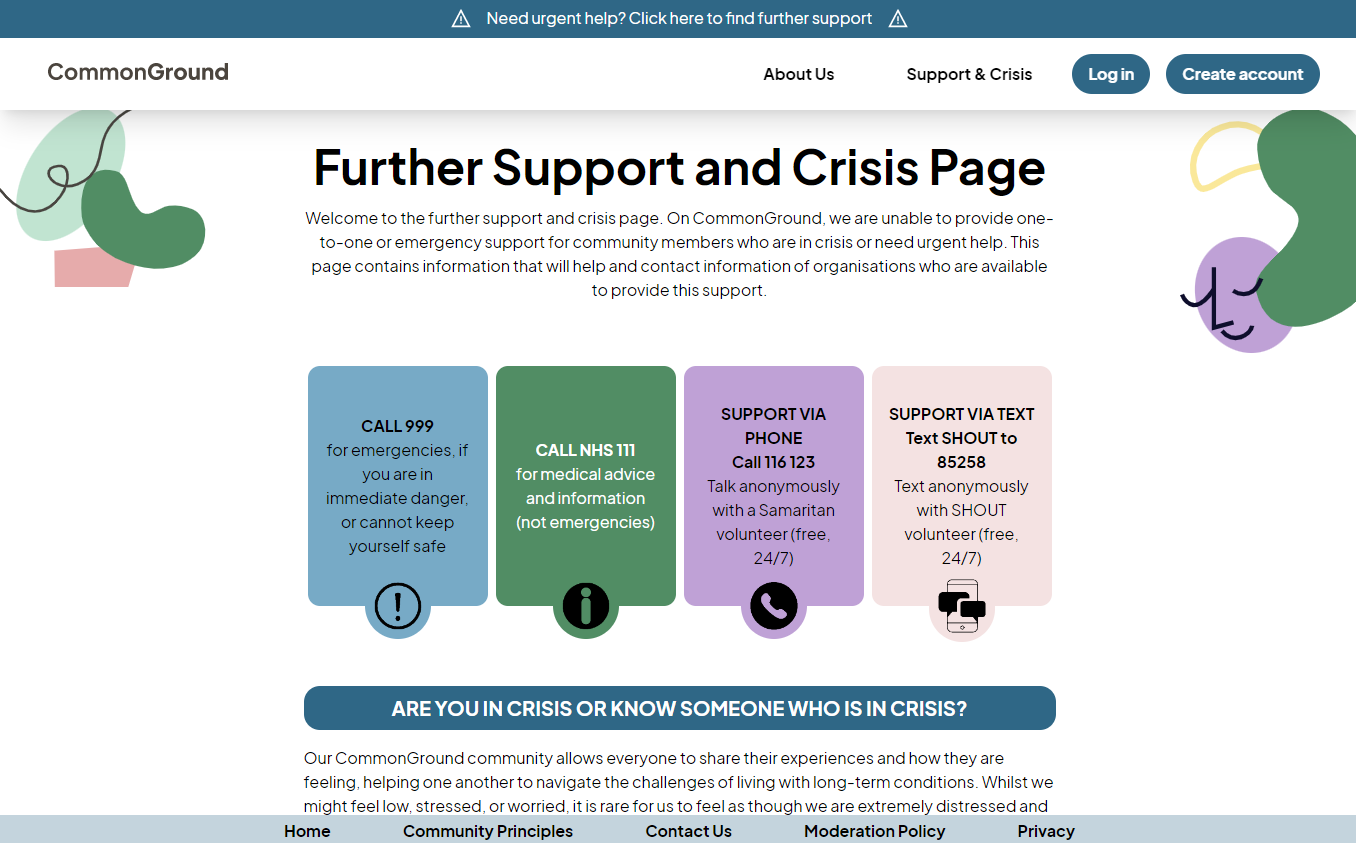


Screenshot 7. Moderation policy page


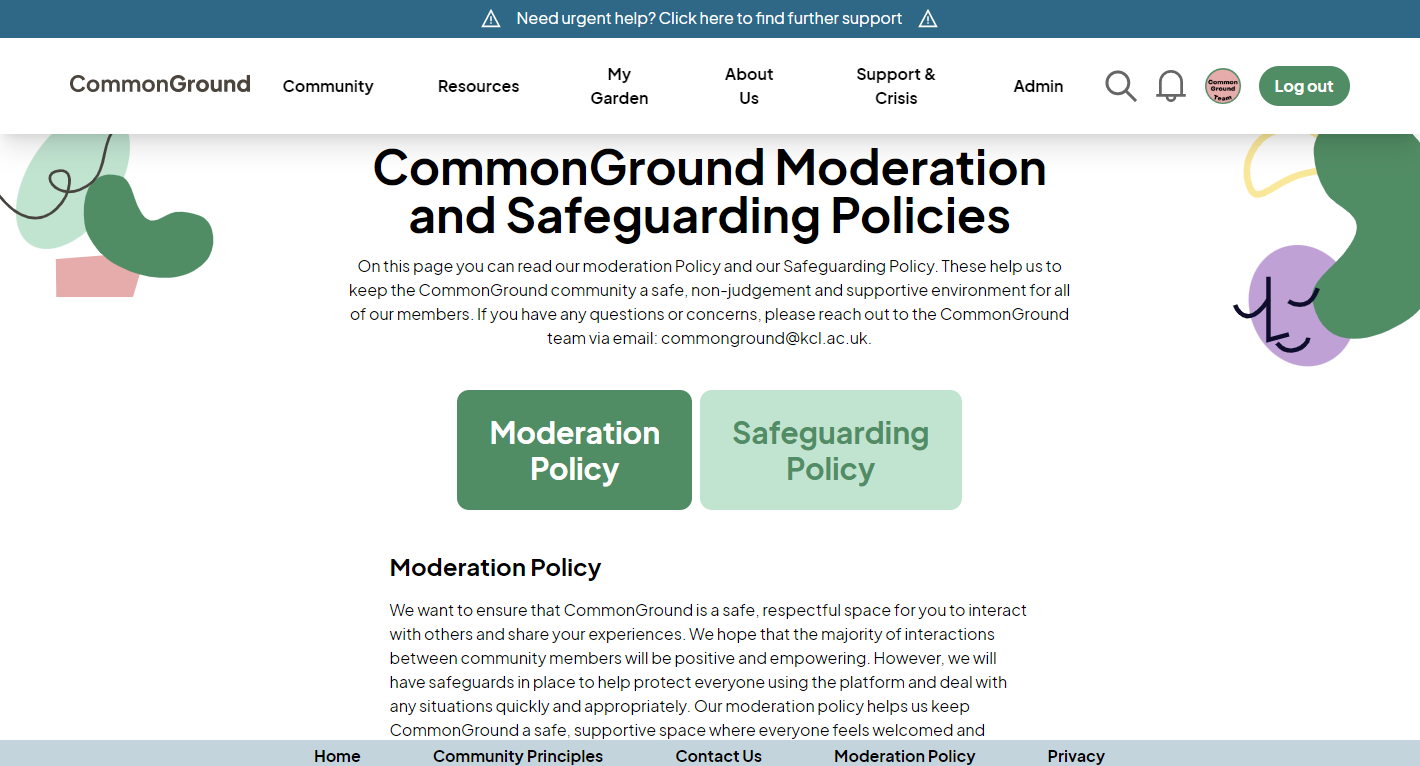


Screenshot 8. Admin panel


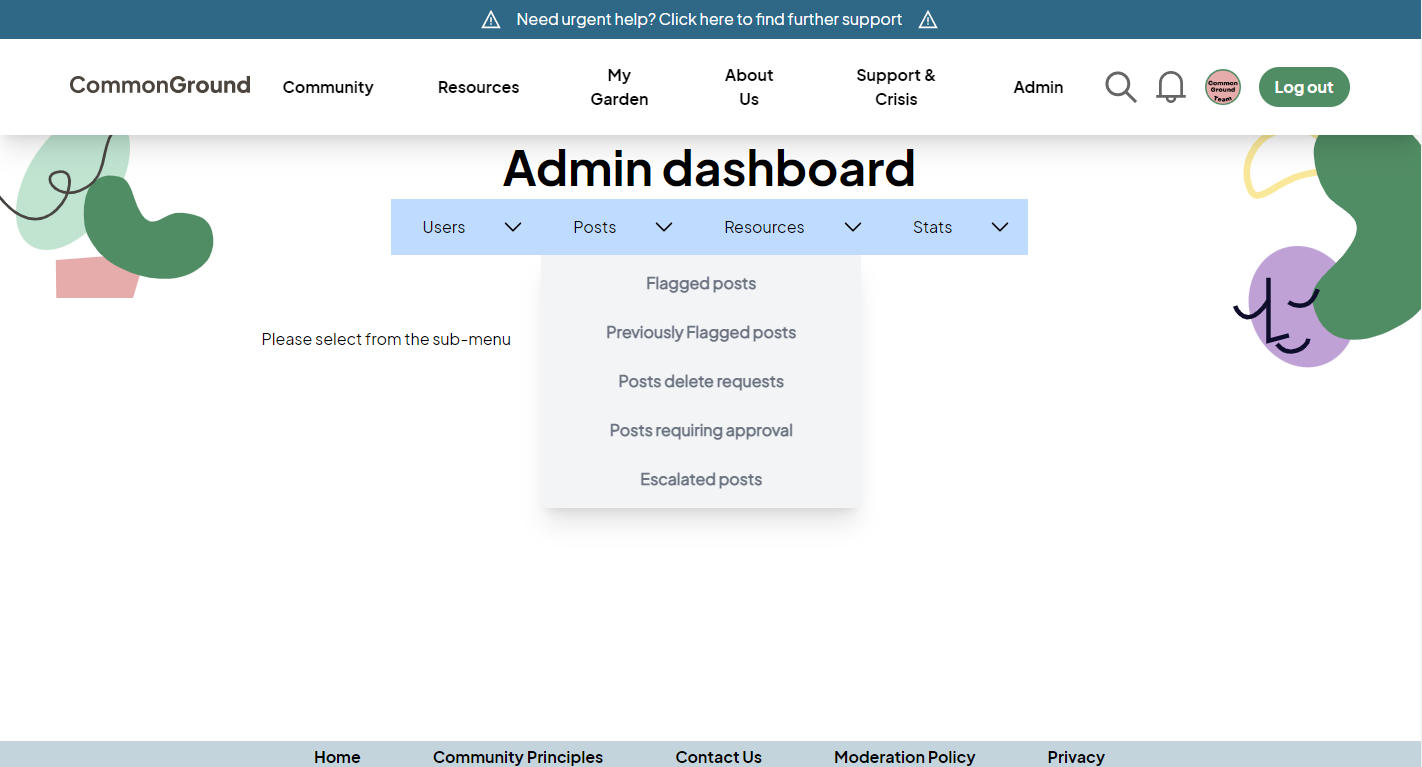

Supplement: Multimedia Appendix 6 [file jmir_v28i1e79666_app6.docx]
